# Application of Passive Sampling to Characterise the Fish Exometabolome

**DOI:** 10.3390/metabo7010008

**Published:** 2017-02-14

**Authors:** Mark R. Viant, Jessica Elphinstone Davis, Cathleen Duffy, Jasper Engel, Craig Stenton, Marion Sebire, Ioanna Katsiadaki

**Affiliations:** 1 School of Biosciences, University of Birmingham, Edgbaston, Birmingham B15 2TT, UK; cathleenireneduffy@yahoo.com; 2 Centre for Environment, Fisheries and Aquaculture Science, Cefas Weymouth Laboratory, Weymouth, Dorset DT4 8UB, UK; jessica.elphinstone-davis@cefas.co.uk (J.E.D.); craig.stenton@cefas.co.uk (C.S.); marion.sebire@cefas.co.uk (M.S.); ioanna.katsiadaki@cefas.co.uk (I.K.); 3 NERC Biomolecular Analysis Facility-Metabolomics Node (NBAF-B), School of Biosciences, University of Birmingham, Edgbaston, Birmingham B15 2TT, UK; J.Engel@science.ru.nl

**Keywords:** DIMS, FT-ICR, bile acid, environmental metabolomics, metabolic footprinting, fish, exogenous

## Abstract

The endogenous metabolites excreted by organisms into their surrounding environment, termed the exometabolome, are important for many processes including chemical communication. In fish biology, such metabolites are also known to be informative markers of physiological status. While metabolomics is increasingly used to investigate the endogenous biochemistry of organisms, no non-targeted studies of the metabolic complexity of fish exometabolomes have been reported to date. In environmental chemistry, Chemcatcher^®^ (Portsmouth, UK) passive samplers have been developed to sample for micro-pollutants in water. Given the importance of the fish exometabolome, we sought to evaluate the capability of Chemcatcher^®^ samplers to capture a broad spectrum of endogenous metabolites excreted by fish and to measure these using non-targeted direct infusion mass spectrometry metabolomics. The capabilities of C18 and styrene divinylbenzene reversed-phase sulfonated (SDB-RPS) Empore™ disks for capturing non-polar and polar metabolites, respectively, were compared. Furthermore, we investigated real, complex metabolite mixtures excreted from two model fish species, rainbow trout (*Oncorhynchus mykiss*) and three-spined stickleback (*Gasterosteus aculeatus*). In total, 344 biological samples and 28 QC samples were analysed, revealing 646 and 215 *m*/*z* peaks from trout and stickleback, respectively. The measured exometabolomes were principally affected by the type of Empore™ (Hemel Hempstead, UK) disk and also by the sampling time. Many peaks were putatively annotated, including several bile acids (e.g., chenodeoxycholate, taurocholate, glycocholate, glycolithocholate, glycochenodeoxycholate, glycodeoxycholate). Collectively these observations show the ability of Chemcatcher^®^ passive samplers to capture endogenous metabolites excreted from fish.

## 1. Introduction

Metabolomics has emerged as a powerful technique to study fish biology, including applications in toxicology [1,2,3,4], nutrition [5,6] and disease [7,8,9]. Further studies have characterised the metabolic changes associated with fundamental processes such as fish embryogenesis [10,11]. These investigations have been conducted across a range of freshwater and marine fish, including a study of the blood biochemistry of whale sharks (*Rhincodon typus*), the world’s largest fish species [12]. Consistent with the large number of metabolomics studies of mammals, in particular humans, the majority of studies have analysed the low molecular weight metabolites in either endogenous tissues or biofluids, i.e., studies of the fish endometabolome [13,14]. Recently, in the context of aquatic toxicology, high sensitivity mass spectrometry based metabolomics approaches have been used to identify xenobiotics and their metabolites, which can bioconcentrate in fish exposed to environmental pollutants [15,16,17]. Such low molecular weight metabolites of exogenous origin are referred to as belonging to the xenometabolome. One further important class of metabolites form what is termed the exometabolome, i.e., the endogenous metabolites that are excreted by organisms into their surrounding environment. In fish biology, a few specific metabolites within the exometabolome have previously been shown to be informative markers of the physiological status of fish, including excreted cortisol, melatonin and sex steroids [18,19,20,21,22]. To date, no non-targeted metabolomics investigations have been reported into the metabolic complexity of fish exometabolomes. Studies of the exometabolomes of cells in culture have, however, been widely reported (also termed metabolic footprinting) and have revealed deep insights into cellular biochemistry [23,24]. In addition the exudates of lower aquatic organisms have recently been investigated [25,26].

The sampling of metabolites excreted from cells into their surrounding media is relatively straightforward, achieved by removing an aliquot of the media for non-targeted metabolomics analysis. The sampling of a broad spectrum of metabolites excreted from fish, typically into a large volume of freshwater or seawater, is much more challenging especially if those metabolites are excreted in low amounts. In the fields of environmental chemistry and toxicology, a number of methods have been developed to extract low concentrations of pollutants from water samples. One such technology is the Chemcatcher^®^ passive sampler, developed over the past 15 years and now used by academic scientists, governmental and environmental agencies and the water industry throughout the world [27]. The Chemcatcher^®^ comprises a polytetrafluoroethylene (PTFE) body into which a 47 mm 3M Empore™ disk is inserted as the receiving phase. The choice of disk depends on the polarity of the pollutants under investigation, with a C18 Empore™ disk (octadecyl phase) for capturing non-polar organics and a SDB-RPS Empore™ disk (comprising a poly(styrenedivinyl-benzene) copolymer that has been modified with sulfonic acid groups to make it hydrophilic) for more polar organics. By deploying a Chemcatcher^®^ within an aqueous environment for several days, it can measure a time-weighted average of the chemicals in that environment. From our review of the tens of publications using Chemcatcher^®^ technology, to date this approach has been used solely to extract organic pollutants and trace metals from water [28,29,30,31]. No studies have been reported that tested this methodology for capturing a broad spectrum of endogenous metabolites excreted by fish, i.e., it has never been applied to the study of the fish exometabolome. The application of such a passive sampler for studying excreted metabolites has many benefits compared to a point sampling strategy, specifically it can overcome the challenges associated with diurnal variation, short term disturbances in metabolite excretion as well as any temporal effects caused by feeding. 

Given the importance of characterising the fish exometabolome, for studies of fish toxicology, nutrition, health and welfare, here we sought for the first time to evaluate the capability of Chemcatcher^®^ passive samplers to capture a broad spectrum of endogenous metabolites excreted by fish, and then to measure the exometabolome using a non-targeted direct infusion mass spectrometry based metabolomics approach [32,33,34]. Given that endogenous metabolites vary considerably in polarity we tested and contrasted the capabilities of two receiving phases, C18 and SDB-RPS Empore™ disks, to capture non-polar and polar organics, respectively. Importantly, these investigations were conducted using real, complex metabolite mixtures excreted from fish, and studies were undertaken on two model fish species, rainbow trout (*Oncorhynchus mykiss*) and three-spined stickleback (*Gasterosteus aculeatus*) to ensure the broad relevance of the results. Salmonids (hence the choice of rainbow trout) represent 99% of aquaculture production in the UK, are used extensively for studying fish disease, and represent a relatively highly domesticated species. In contrast, three-spined stickleback are far less domesticated than trout and have no commercial interest. Importantly they represent a different family (*Gasterosteidae*), potentially adding diversity into the two exometabolomes studied here. Furthermore, fish were studied over a 4-week period to assess any variations in their exometabolomes over time. The original aim of these studies was to apply non-targeted metabolomics, sampled as described above, for the discovery of fish welfare markers. In this respect the fish were kept under different housing environments and husbandry conditions. However, every attempt to analyse the samples in this light was unfruitful. As such, multivariate statistical analyses were used to compare and contrast the exometabolome fingerprints excreted by the two fish species and captured on the two receiving phases, across four time points, as proof of principle of this method for studying the exometabolome.

## 2. Results

### 2.1. Effect of Empore™ Disk and Fish Species on the Measured Exometabolome

**Figure 1:**
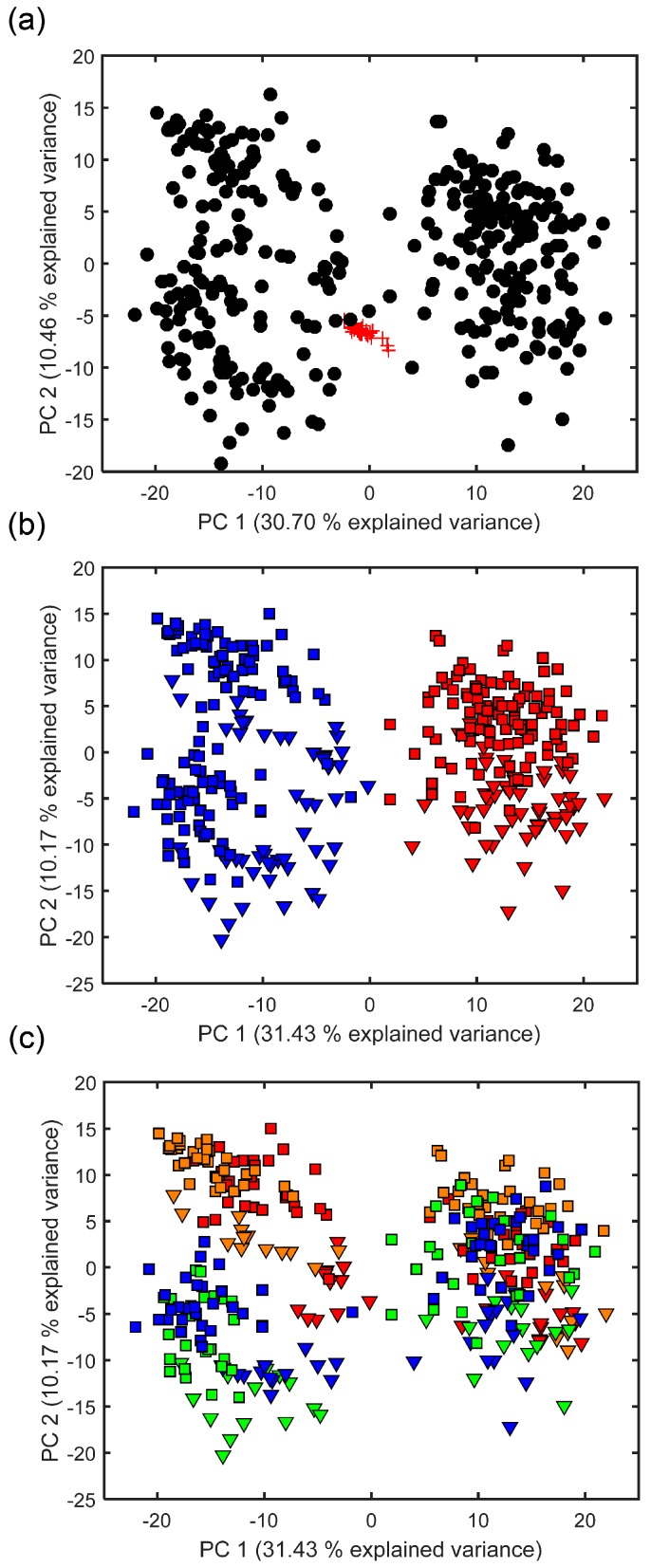
Principal components analysis (PCA) scores plots from analysis of the fish-derived metabolites that were extracted using Chemcatcher^®^ passive samplers and measured via direct infusion Fourier transform ion cyclotron resonance (FT-ICR) mass spectrometry metabolomics. (**a**) Analysis of both the quality control (QC) and biological samples, highlighting the high technical reproducibility of the metabolomics measurements of the 28 QC samples. Key: QC (red) and biological samples (black); (**b**) Biological samples only, labelled so as to highlight any differences in the exometabolomes that were excreted by rainbow trout and three-spined stickleback that were captured onto C18 (non-polar) and styrene divinylbenzene reversed-phase sulfonated (SDB-RPS) (polar) Empore™ disks. Key: C18 disk (blue), SDB-RPS disk (red), stickleback (square) and trout (triangle); (**c**) Biological samples only, labelled to highlight any differences in the exometabolomes excreted across the 4-week investigation. Multivariate analysis of variance (MANOVA) of PCA scores for three-factor model, *p* < 1.0 × 10^−3^ for type of disk, fish species and sampling time; two-way interaction of disk x time was significant (*p* < 1.0 × 10^−3^), interaction of disk x species was significant (*p* = 3.7 × 10^−5^), and interaction of species x time was not significant (*p* = 6.4 × 10^−1^). Key: metabolites captured in week 1 (red), week 2 (orange), week 3 (green) and week 4 (blue), for stickleback (square) and trout (triangle).

Following extensive data processing, direct infusion FT-ICR mass spectra of the excreted fish metabolites that were captured by the Empore™ disks yielded a single data matrix of 344 biological samples and 28 QC samples, comprising of 974 unique *m*/*z* values. This relatively low number of peaks detected, compared to DIMS metabolomics studies of biofluids and tissue extracts that are more routinely investigated in metabolomics studies [35], reflects the relatively low concentrations of metabolites in the Chemcatcher^®^ derived samples. PCA was used initially to assess the technical reproducibility of this mass spectrometry metabolomics dataset. The tight clustering of 25 of the 28 QC samples within the PCA scores plot confirms the high technical reproducibility (Figure 1a). The QC samples were then removed from the dataset and a second PCA conducted to highlight any metabolic differences between the use of C18 and SDB-RPS Empore™ disks, between the rainbow trout and three-spined stickleback, and between the four time points. Figure 1b clearly highlights that the primary effector is the type of Empore™ disk used, with separation of the C18 and SDB-RPS samples along PC1 and accounting for 31.43% of the variance within the dataset. The fish exometabolomes captured by the SDB-RPS disks clustered somewhat more tightly than those obtained from the C18 disks. Figure 1b also reveals that the two fish species might excrete somewhat differing exometabolomes, with the rainbow trout samples occurring at slightly higher PC2 values. This difference between the two species groups may, however, originate for a number of reasons, including genetic, environmental or simply due to differences in the fish biomasses, as discussed below. Figure 1c shows the same PCA scores plot, but is labelled differently in order to highlight any potential variation in the fish exometabolomes across the four sampling times. The cause of the greater dispersion of the C18 disks (relative to SDB-RPS disks) is now evident, with the C18 measured exometabolome changing somewhat between weeks 1–2 and weeks 3–4, along the PC2 axis.

### 2.2. Comparison of Putatively Annotated Metabolites Captured on Empore™ Disks

Given the findings above, in particular the dominant effect of the type of Empore™ disk, the dataset was separated into four blocks (C18 trout, C18 stickleback, SDB-RPS trout, SDB-RPS stickleback) to facilitate a more detailed examination of each class. Following this reprocessing, each class comprised of multiple biological samples and a single peak (*m*/*z*) list. The total of four peak lists were annotated with empirical formula(e) and putative metabolite names using MI-Pack and KEGG (Tables S1–S4); this tabulated data comprises of *m*/*z* value, median peak intensity, empirical formula(e) (i.e., C_c_H_h_N_n_O_o_P_p_S_s_), corresponding ion form, theoretical ion mass (Da), mass measurement error (ppm) between the measured and theoretical values, and any putative metabolite names assigned using the KEGG_COMPOUND terminology. As justified below (see Section 3), we additionally focused on the identification of selected bile acids in the FT-ICR mass spectra as these metabolites were known to be present in the trout metabolome [36] and hence would serve to strengthen the evidence that the methodology is performing as anticipated. For example, taurocholate (the taurine-conjugated bile acid cholate) was measured in extracts from both the C18 and SDB-RPS disks, detected at *m*/*z* 514.28473 as the [M − H]^−^ ion form of C_26_H_45_NO_7_S, the exact mass measurement error was only 0.64 ppm (between theoretical and measured), the ^13^C isotope pattern correctly predicted an ca. 25.7 carbon atom containing metabolite, and the ^34^S isotope pattern correctly predicted a ca. 1.2 sulfur atom containing chemical. In addition, MS/MS (CID) confirmed its identity.

**Figure 2:**
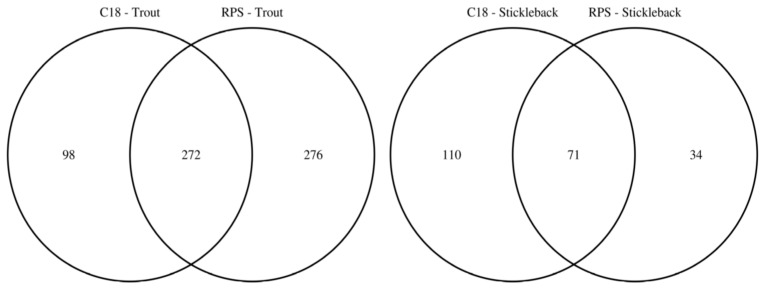
Venn diagrams highlighting the complementarity of C18 and SDB-RPS Empore™ disks in a Chemcatcher^®^ passive sampler, by showing the numbers of putatively annotated metabolites captured from the water by each type of disk for rainbow trout and three-spined stickleback. Relatively few metabolites are captured by both receiving phases.

**Figure 3:**
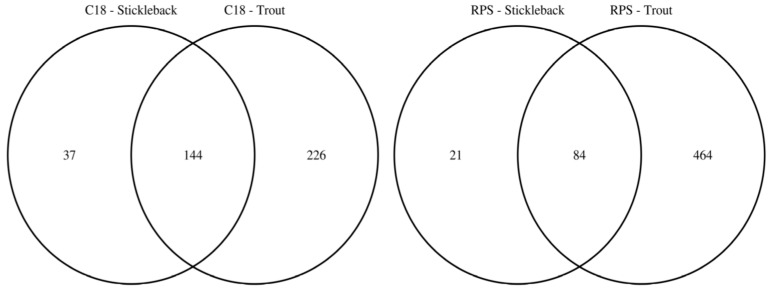
Venn diagrams highlighting the larger number of exometabolic peaks arising from rainbow trout versus three-spined stickleback, by showing the numbers of putatively annotated metabolites excreted from each fish species using C18 and SDB-RPS Empore™ disks in a Chemcatcher^®^ passive sampler. The majority of peaks measured from stickleback are also observed in the trout datasets.

To facilitate the comparison of the putatively annotated metabolites measured in each of the four classes, Venn diagrams were constructed using a 1 ppm *m*/*z* tolerance. Figure 2 compares the metabolites captured by the C18 and SDB-RPS Empore™ disks, separately for each fish species. For trout (Figure 2a), 370 peaks were detected in the mass spectra of the C18 disks, while approximately 50% more (548 peaks) were measured from the SDB-RPS disks. Of these, 272 peaks were shared between the two types of disk, equating to 73.5% and 49.6% of the peaks in the C18 and SDB-RPS trout datasets. Fewer metabolites were observed in the stickleback datasets (Figure 2b), with 181 peaks detected using the C18 disk, and 105 peaks measured from the SDB-RPS disks. Of these, 71 peaks were shared between the two types of disk, equating to 39.2% and 67.6% of the peaks in the C18 and SDB-RPS stickleback datasets. Further Venn diagrams were constructed to compare the measured exometabolomes of the trout and stickleback, separately for each type of Empore™ disk. For the C18 receiving phase (Figure 3a), 370 peaks were detected in the trout dataset, while only 181 peaks were measured from the stickleback. Of these, 144 peaks were shared between the two fish species, which accounted for the majority (79.6%) of the stickleback dataset. A similar finding was observed for the SDB-RPS receiving phase (Figure 3b), with far more peaks detected in the trout (548 peaks) compared to the stickleback (105 peaks). Of these, 84 peaks were shared between the two fish species, again representing the majority (80.0%) of the stickleback dataset.

### 2.3. Effect of Sampling Time on the Measured Fish Exometabolome

**Figure 4:**
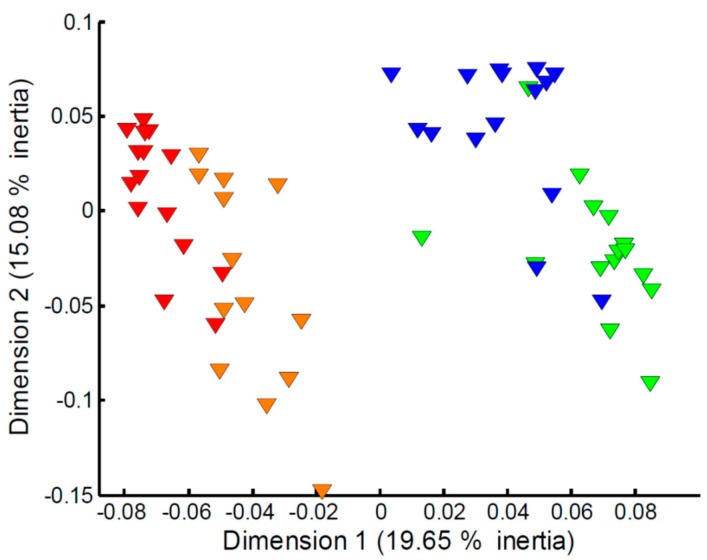
Multiple correspondence analysis of the metabolites captured on the SDB-RPS receiving phase in the trout tanks and measured via mass spectrometry metabolomics. Metabolite data were represented in an indicator matrix (presence/absence). MANOVA of scores, *p* < 1.0 × 10^−3^, and all pairwise comparisons between four time points, *p* ≤ 1.6 × 10^−3^). Key: metabolites captured in week 1 (red), week 2 (orange), week 3 (green) and week 4 (blue).

Focusing on the apparent effect of sampling time on the measured exometabolomes (Figure 1c), in particular for the Empore™ C18 disk, further multivariate analyses of the four classes (C18 trout, C18 stickleback, SDB-RPS trout, SDB-RPS stickleback) were undertaken. However, large differences in the numbers of missing values at weeks 1 and 2 compared to weeks 3 and 4 were observed. As a result, imputation of the missing values and subsequent PCA analysis was deemed too unreliable to study the effect of sampling time. Instead we employed MCA, a multivariate statistical approach that focuses on the presence or absence of peaks, and hence the imputation of missing values is not needed. Figure 4 shows the MCA scores plot for the metabolites captured on the SDB-RPS receiving phase in the trout tanks. The effect of sampling time is clearly evident, with Empore™ disks added to the tanks (and subsequently recovered) in weeks 1–2 showing a large difference to those added to the tanks in weeks 3–4. The equivalent plots for the three other datasets, highlighting similar temporal effects, are presented in Figure S1. 

## 3. Discussion

The results presented here, for the first time, show that a broad spectrum of endogenous metabolites excreted from fish can be captured using Empore™ disks and Chemcatcher^®^ passive samplers and subsequently measured using a non-targeted direct infusion mass spectrometry based metabolomics approach, the latter relying upon nanoelectrospray ionisation to achieve high analytical sensitivity. Rigorous data processing was applied, including multiple signal filtering steps, to ensure that the peaks reported here arise from the fish and not from food, solvents, plasticisers or other contaminants from the extraction procedure [37,38]. This analytical and computational approach was able to detect metabolites from two species of fish, highlighting its generality. A total of 646 unique peaks were detected from trout (370 from C18 disks, 548 from SDB-RPS, less the overlap), while only 215 unique peaks were detected from stickleback (181 from C18 disks, 105 from SDB-RPS, less the overlap); the greater number of peaks in the trout datasets is also clearly visible in Figure 3. This observation can be rationalised by considering the biomass of fish used in each experimental replicate. For trout, the starting biomass was ca. 450 g per 40 L aquarium, equating to ca. 10 g/L. The starting biomass for stickleback was ca. 4 g per 10 L aquarium, or 0.4 g/L. Hence each trout aquarium comprised of ca. 20 times as much biomass than for the sticklebacks; which naturally excreted a greater amount of metabolites into the water. In addition, the stickleback aquaria were also supplied with a biological filter, potentially reducing further the metabolite abundance in the water. As well as rationalising the overall numbers of peaks detected for the two fish species, these differences in biomass may also explain the separation of the species in the PCA scores plots (Figure 1). While a consideration of biomass rationalises the stronger metabolic signals from the trout experiments, it is important to note that even the much smaller stickleback generated sufficient metabolites for their exometabolome to be detectable by nanoelectrospray DIMS. Encouraged by this finding, we have also investigated and found that metabolites excreted from zebrafish (*Danio rerio*) can also be captured and measured using an SDB-RPS receiving phase (data not shown), which offers a powerful methodology to this model organism’s research community [39,40].

Given that endogenous metabolites are known to vary considerably in polarity, the capabilities of two receiving phases were evaluated and contrasted, specifically C18 and SDB-RPS Empore™ disks to capture non-polar and polar metabolites, respectively. Not surprisingly, the chemistry of the receiving phase was shown to have the largest effect on which metabolites were captured and measured (Figure 1b). From the more detailed analysis presented in Figure 2 it can be argued that both disks should be used when studying the fish exometabolome. Specifically, only 42.1% of the peaks are captured by both C18 and SDB-RPS disks for trout and only 33.0% by both disks for stickleback, indicating the importance of each unique phase chemistry. Should logistics constrain a particular study to only one receiving phase, however, the higher intensity trout dataset indicates that the SDB-RPS disk is the preferred choice.

Consistent with DIMS metabolomics studies of non-mammalian organisms, some of the peaks measured in the FT-ICR analysis of the Empore™ disk extracts were putatively annotated with a metabolite name, some assigned an empirical formula, while many peaks were not present in the KEGG database and hence could not be identified [35]. In this investigation the majority of peaks that were annotated with a metabolite name were done so at level 2 of the Metabolomics Standards Initiative reporting requirements [41]. However, we additionally interpreted the mass spectrometry data of some metabolites that were known, or at least anticipated, to be present in the fish exometabolome as a form of validation for this analytical and computational approach. Bile acids were selected as their composition in rainbow trout is well documented [36]. Furthermore there is some evidence that bile acids serve as pheromones [42], for which their excretion from the fish into the surrounding water is required, providing ideal target compounds for our methodology. Considering this existing literature, cholic acid was determined previously to be the primary bile acid with chenodeoxycholic acid in lower abundance. The bile acids were reported to be mostly taurine conjugated and to a lesser degree glycine conjugated, forming glycocholic acid. Sulfation of these compounds was also documented, e.g., ca. 5% of taurocholate was sulfated, as were trace amounts of cholic and glycocholic acids [36]. In the current study, several such metabolites were detected and annotated including: chenodeoxycholic acid (C_24_H_40_O_4_ as the [M + Cl]^−^ ion); dehydrocholic acid (C_24_H_34_O_5_ as the [M + Cl]^−^ ion); glycolithocholic acid (C_26_H_43_NO_4_ as the [M + Na-2H]^−^ ion); glycochenodeoxycholic acid or glycodeoxycholic acid (C_26_H_43_NO_5_ as the [M + Na-2H]^−^ ion); glycocholic acid (C_26_H_43_NO_6_ as the [M + Na-2H]^−^ ion); taurocholate (C_26_H_45_NO_7_S as the [M − H]^−^ ion) and glycochenodeoxycholic acid 7-sulfate (C_26_H_43_NO_8_S as the [M − H]^−^ ion). Collectively these observations show the ability of the Chemcatcher^®^ passive samplers to capture endogenous metabolite mixtures excreted from fish, representing the first measurements of the rainbow trout and three-spined stickleback exometabolomes. 

## 4. Materials and Methods 

### 4.1. Fish Husbandry

Two common laboratory fish species (rainbow trout, *Oncorhynchus mykiss*; three-spined stickleback, *Gasterosteus aculeatus*) were used in these studies. The fish were kept under three housing conditions; standard, sub-standard, and supra-standard for the duration of the test, with constant or episodic modifications to husbandry techniques and the visual and acoustic environments. Since these conditions did not result in any differences within the metabolite profiles, they are not described here. All fish used in the study were reared in-house. Fertilised rainbow trout eggs (eyed ova; all female) were obtained from a commercial supplier (Troutlodge, Isle of Man, UK) in April 2013 and were reared following standard practices in the Cefas breeding facility. The three-spined sticklebacks were obtained from the Cefas Weymouth laboratory bred colony (well established over 17 years). Fish from the breeding facility were randomly allocated to their experimental units. Each tank/aquarium constituted one experimental unit/replicate; the number of fish per replicate was determined by standard practice and in-house expertise based on optimal social dynamics for each species. The tanks/aquaria were spatially distributed in a way that prevented any potential impact of housing modifications (i.e., noise) on non-target replicates. Rainbow trout (30 females per tank only, 15 g at start of experiment) were maintained in 15 circular, opaque 40 L fibreglass tanks (equivalent to biomass of ca. 450 g per tank). Each tank was supplied with fresh water (temperature 12 ± 1 °C, salinity 0‰, dissolved oxygen >7.0 mg/L) at a flow rate of 0.5 L/min, and provided with an airlift “streamer” to create a clockwise current. They were fed a commercial diet (Nutra Parr 1.8, Skretting Fish Feeds) at the recommended feeding rate, three times daily. The three-spined stickleback (10 mixed sex fish per aquarium, 0.4 g at start of experiment) were maintained in 30 rectangular, transparent 10 L glass aquaria (equivalent to biomass of ca. 4 g per aquarium). Each aquarium contained an under gravel biological filter, and was supplied with fresh water (temperature 13 ± 1 °C, salinity 0‰, dissolved oxygen >7.0 mg/L) at a flow rate of 0.04 L/min. The sticklebacks were fed *Artemia* (cysts obtained from Brine Shrimp Direct) and a commercial diet of frozen mosquito larvae (mini bloodworm, Gamma slice) and, at the recommended feeding rate, twice per day. 

All laboratory studies were undertaken under the authority of a UK Home Office licence (PPL 3003122) in accordance with national regulations and were approved by the Cefas local Animal Welfare and Ethical Review Body. Fish were monitored daily by research staff and animal technicians; any abnormal behaviour or appearance were noted. During the first week of the experimental phase two unexpected incidents occurred in two separate rainbow trout tanks: one fish displayed poor buoyancy (it was humanely killed) and another was found dead after being trapped behind the air streamer (the streamer layout was subsequently modified). Consequently, the number of rainbow trout was reduced to 28 across all tanks to ensure comparability of the ensuing metabolite profiles. At the end of the four-week experimental period all fish were killed by terminal anaesthesia (MS222). 

### 4.2. Capture of Fish Exometabolomes Using Chemcatcher^®^ and Empore™ Disks

The receiving phase disks used to capture the non-polar and polar exometabolomes were C18 and SDB-RPS Empore™ disks, respectively. They were pre-conditioned according to the manufacturer’s instructions. Briefly, the C18 disks were washed with 20 mL methanol followed by one wash of HPLC grade water (via a manifold), ensuring the disks were not dry at any point. The SDB-RPS disks were washed in the same way using 10 mL of acetone, followed by 10 mL of isopropanol, 10 mL of methanol, and finally 10 mL of HLPC grade water. All solvents were purchased from Sigma-Aldrich (Poole, Dorset, UK). The disks were then mounted into the Chemcatcher^®^ devices according to the manufacturer’s instructions and were deployed within a polystyrene container positioned in the outflow of each tank/aquarium. Fish were maintained as described above, for four weeks. The following sampling protocol was designed after two pilot studies: time averaged collection from days 1–4 (disks labelled as week 1), collection from days 8–11 (week 2), from days 15–18 (week 3), and from days 22–25 (week 4). Each week the Empore™ disks were removed, air dried with the use of a manifold, wrapped in aluminium foil and stored at −80 °C until extraction. 

### 4.3. Extraction of Receiving Phase Empore™ Disks

Disks were removed from the −80 °C freezer and placed in a methanol (HPLC grade) rinsed 7-mL glass vial. To each of the C18 disks, 3 mL of ice-cold methanol was added and sonicated for 20 min. Two mL of extracted solvent was removed into a clean Eppendorf tube and dried in a centrifugal concentrator (Thermo Savant, Holbrook, NY, USA) at 35 °C. To each of the SDB-RPS disks, 3 mL of ice-cold 70/30 methanol/water (HPLC grade; pH set to ca. 9.5 by addition of ammonium hydroxide) was added and then sonicated for 20 min. Two mL of the extracted solvent was removed and dried, as above. All dried extracts were stored at −80 °C until metabolomics analysis.

### 4.4. Direct Infusion Mass Spectrometry Metabolomics 

Direct infusion mass spectrometry (DIMS) based metabolomics was performed on a 7-Telsa Fourier transform ion cyclotron resonance (FT-ICR) mass spectrometer (LTQ-FT Ultra, Thermo Fisher Scientific, Bremen, Germany) with a chip-based Triversa direct infusion nanoelectrospray source (Advion Biosciences, Ithaca, NY, USA). Dried extracts were reconstituted in 40 μL of 10 mM ammonium acetate in 80/20 methanol/water, vortexed (30 s), and centrifuged (5 min) to remove any particular matter. They were then analysed in negative ion mode in a controlled-randomised sequence different from the extraction sequence, with each sample analysed as three technical replicates. Quality control (QC) samples (derived from a pool of the extracted samples) were analysed repeatedly at the start, end, and equidistantly throughout the measurement of the biological samples by DIMS. Data was acquired at a nanoelectrospray voltage of −1.5 kV, 0.9 psi backing pressure, and at a nominal resolution of 100,000 (at *m*/*z* 400) in several SIM (selected ion monitoring) windows of 200 Da width, from *m*/*z* 70 to 800 [32,33]. Mass spectra were processed and analysed statistically as described below.

### 4.5. Data Processing and Peak Annotation

Metabolomics data were processed using the SIM-stitching algorithm [34,43] using in-house Matlab scripts. High quality reproducible data were achieved by implementing a series of peak filtering algorithms: first, peaks within the FT-ICR mass spectra were picked using an established signal to noise ratio threshold of 3.5:1. A “replicate filter” was applied such that only peaks in two (or more) of the three technical replicates (per sample) were retained, and then a “sample filter” was applied to all the mass spectra to retain only those peaks in >50% of all of the samples. These strict criteria are required to minimise noise within the metabolomics datasets [43]. Further filtering was then applied to ensure that any peaks retained in the biological data matrix were derived from the fish exometabolome and not from the extraction procedure nor from the food within the fish tank/aquaria. To achieve this “blank filtering”, Empore™ disks that had been placed in the fish tank/aquaria (*n* = 4 disks per type of tank/aquaria, i.e., per species) and exposed to food but not to any fish were extracted using the same protocols as described above. These “blank samples” were analysed using the same direct infusion mass spectrometry method and any peaks observed (i.e., from food, solvents, plasticisers or other contaminants from the extraction procedure) were subtracted from the mass spectra of the disk extracts that had been exposed to the fish; an established threshold was used such that all peaks in the extract blank were removed from the biological data matrix except those exceeding a minimum sample-to-blank intensity ratio of 10 (which are deemed to be predominantly of biological origin). Next, any peak remaining in the dataset with a percentage of missing values (across the biological samples) greater than 60% was removed, again a standard step in the workflow to remove problematic samples prior to statistical analysis. The resulting peak intensity matrix described the fish exometabolome. The intensity matrix was normalised using the probabilistic quotient normalisation algorithm [44], missing values were imputed using a KNN algorithm [45] and then the data were transformed using the generalised logarithm [46] to stabilise the technical variance across the measured peaks prior to analysis using multivariate statistics. As justified in the Results section, the single data matrix comprising all samples was subsequently separated into four classes, one for each combination of fish species and Empore™ disk (trout-C18, trout-SDB-RPS, stickleback-C18, stickleback-SDB-RPS). This was achieved using a 50% “sample filter” for each of the four classes and a batch correction algorithm that further enhanced the data quality by removing peaks that were not present in all the QC samples [47]. Finally, peaks in the mass spectra were annotated (to Metabolomics Standards Initiative, level 2 [41]) and putative empirical formulae calculated based upon accurate mass measurements and isotope patterns, using both MI-Pack software [48] and by searching the KEGG database (3 ppm *m*/*z* tolerance; [49]). Further identification of selected metabolites was performed by MS/MS fragmentation (collision induced dissociation, CID) using the FT-ICR mass spectrometer.

### 4.6. Statistical Analyses of Metabolomics Measurements

Principal components analysis (PCA) was used to assess the overall metabolic similarities and differences between the samples in an unbiased manner using Matlab (version 7.8; The MathsWorks, Natick, MA, USA). Specifically, PCA was conducted to investigate any differences between the exometabolomes captured on C18 versus SDB-RPS Empore™ disks, differences between the metabolites excreted from rainbow trout versus three-spined stickleback, and variation in the metabolites captured over the 4-week investigation period. Multivariate analysis of variance (MANOVA) was used to determine the significance of the differences observed in the PCA scores plots. The p-value of post-hoc pairwise comparisons was corrected by the Bonferroni-Holm procedure. In addition, multiple correspondence analysis (MCA) was used to assess the effect of sampling time on the fish exometabolome. For this purpose, the metabolite data for each of the four classes was represented as an indicator matrix highlighting for each sample whether a peak was present or absent. Each of the four indicator matrices was used as an input to MCA. Significance by MANOVA was carried out as described for PCA above.

## Supplementary Materials

The following are available online at www.mdpi.com/2218-1989/7/1/8/s1, Figure S1: Multiple correspondence analyses of the metabolites captured on the Empore™ receiving phases in the fish aquaria and measured via mass spectrometry metabolomics, Table S1: Putative annotations of metabolites measured in the C18 trout class using FT-ICR mass spectrometry, Table S2: Putative annotations of metabolites measured in the SDB-RPS trout class using FT-ICR mass spectrometry, Table S3: Putative annotations of metabolites measured in the C18 stickleback class using FT-ICR mass spectrometry, Table S4: Putative annotations of metabolites measured in the SDB-RPS stickleback class using FT-ICR mass spectrometry.
