# Supplementary material for: Application of Passive Sampling to Characterise the Fish Exometabolome
Source: Metabolites. 2017 Feb 14;7(1):8. doi: 10.3390/metabo7010008 (PMC5372211; doi:10.3390/metabo7010008)
Supplement: Supplementary file 1 [file metabolites-07-00008-s001.pdf]

Supplementary Materials: Application of Passive Sampling to Characterise the Fish Exometabolome

Mark R. Viant, Jessica Elphinstone Davis, Cathleen Duffy, Jasper Engel, Craig Stenton, Marion Sebire and Ioanna Katsiadaki

Table S1. Putative annotations of metabolites measured in the C18 trout class using FT-ICR mass spectrometry.

m/z	Median Intensity	Empirical Formula	Ion Form	Theoretical Mass (Da)	Mass Error (ppm)	KEGG_COMPOUND
122.97568	3.89E+03	$C_2H_4O_4S$	[M-H]⁻	122.97576	−0.61	[‘Sulfoacetaldehyde’]
122.97568	3.89E+03	O_2S	[M + HAc-H]⁻	122.97576	−0.61	[‘Sulfur dioxide’]
133.02943	2.03E+03	C_6H_2	[M + HAc-H]⁻	133.02950	−0.55	[‘Triacetylene’]
133.02943	2.03E+03	$C_8H_6O_2$	[M-H]⁻	133.02950	−0.55	[‘NA’]
138.97059	2.59E+03	$C_2H_4O_5S$	[M-H]⁻	138.97067	−0.58	[‘Sulfoacetate’]
162.96550	8.80E+02	$C_2H_6O_4S$	[M + (³⁷Cl)]⁻	162.96513	2.25	[‘2-Hydroxyethanesulfonate’, ‘Dimethyl sulfate’]
176.92820	5.78E+03	0				0
186.03122	1.36E+04	0				0
187.02367	7.65E+03	$C_6H_6N_4O_2$	[M + Na-2H]⁻	187.02374	−0.4	[‘1-Methylxanthine’, ‘3-Methylxanthine’, ‘7-Methylxanthine’]
193.03546	4.85E+03	$C_{10}H_8N_2$	[M + (³⁷Cl)]⁻	193.03520	1.35	[‘3-Indoleacetonitrile’]
193.03546	4.85E+03	$C_4H_6O_5$	[M + HAc-H]⁻	193.03538	0.42	[‘(R)-Malate’, ‘(S)-Malate’, ‘3-Dehydro-L-threonate’, ‘Malate’]
193.03546	4.85E+03	$C_6H_{10}O_7$	[M-H]⁻	193.03538	0.42	[‘2-Dehydro-D-galactonate’, ‘2-Keto-D-gluconic acid’, ‘3-Dehydro-L-gulonate’, ‘5-Dehydro-D-gluconate’, ‘D-Fructuronate’, ‘D-Galacturonate’, ‘D-Glucuronate’, ‘D-Glucuronic acid’, ‘D-Mannuronate’, ‘D-Tagaturonate’, ‘Galacturonic acid’, ‘L-Guluronic acid’, ‘L-Iduronic acid’, ‘beta-D-Glucopyranuronic acid’]
200.04688	5.54E+03	0				0
208.02869	7.11E+02	0				0
208.84540	5.64E+03	0				0

210.84244	8.32E+03	0				0
212.83949	3.97E+03	0				0
214.83663	1.29E+03	0				0
216.07933	1.26E+03	$C_{10}H_{15}NO_2$	[M + Cl]⁻	216.07968	−1.62	[‘NA’, ‘NA’, ‘NA’]
216.07933	1.26E+03	$C_{11}H_{17}NO$	[M + K-2H]⁻	216.07962	−1.34	[‘(+)-*N*-methylpseudoephedrine’, ‘NA’, ‘Mexiletine’, ‘Tecomine’]
218.87421	2.14E+03	0				0
267.14387	1.20E+05	0				0
293.17939	2.53E+06	0				0
294.18275	4.35E+05	$C_{13}H_{21}N_3O$	[M + HAc-H]⁻	294.18232	1.48	[‘Procainamide’]
295.17519	9.93E+04	0				0
308.87607	3.53E+04	0				0
309.17432	2.63E+06	$C_{17}H_{26}N_2O$	[M + Cl]⁻	309.17391	1.31	[‘Ropivacaine’, ‘Sauroxine’, ‘alpha-Obscurine’]
309.17432	2.63E+06	$C_{18}H_{20}N$	[M + HAc-H]⁻	309.17343	2.89	[‘*cis*-*N*-Methyl-(*S*)-7,8,13,14-tetrahydroprotoberberine’]
309.17821	6.23E+04	0				0
310.17768	4.07E+05	0				0
310.87318	3.31E+04	0				0
314.89310	5.36E+04	0				0
321.21070	8.06E+05	0				0
322.21405	1.35E+05	$C_{15}H_{25}N_3O$	[M + HAc-H]⁻	322.21362	1.35	[‘NA’, ‘Triapenthenol’]
323.10676	9.88E+04	$C_{15}H_{20}N_2O_4S$	[M-H]⁻	323.10710	−1.06	[‘Acetohexamide’, ‘Chromanol 293B’, ‘HMR1556’]
323.10676	9.88E+04	$C_{18}H_{16}N_4$	[M + Cl]⁻	323.10690	−0.43	[‘NA’]
323.20652	3.57E+04	0				0
332.92052	1.70E+05	$C_{14}H_4N_2O_2S_2$	[M + K-2H]⁻	332.92003	1.48	[‘Dithianon’]
333.00776	2.94E+04	0				0
337.20564	2.72E+06	0				0
337.20998	1.70E+05	0				0
338.20899	4.97E+05	0				0
343.01720	2.50E+04	0				0
343.16841	6.25E+04	$C_{18}H_{28}O_4$	[M + Cl]⁻	343.16816	0.72	[‘5-O-Methylembelin’, ‘Soraphen O’]

343.16841	6.25E+04	$C_{19}H_{30}O_3$	[M + K-2H]⁻	343.16810	0.9	['3alpha,11beta-Dihydroxy-5alpha-androstane-17-one', '3alpha,11beta-Dihydroxy-5beta-androstane-17-one', '3beta-Hydroxy-D-homo-17a-oxa-5alpha-androstan-17-one', 'Androst-5-ene-3beta,17beta,19-triol', 'Dihydromonacolin L', 'Oxandrolone']
343.16841	6.25E+04	$C_{19}H_{30}OS$	$[M + (^{37}Cl)]^-$	343.16819	0.65	['NA']
347.15114	1.61E+05	$C_{18}H_{16}N_4$	$[M + HAc-H]^-$	347.15135	−0.6	['NA']
349.00266	2.17E+04	0				0
350.12572	8.89E+04	0				0
353.05252	7.41E+04	0				0
353.20053	4.45E+06	0				0
354.20395	7.69E+05	0				0
354.20838	5.46E+04	$C_{20}H_{25}NO$	$[M + HAc-H]^-$	354.20747	2.58	['NA', 'NA', 'NA']
354.20838	5.46E+04	$C_{20}H_{33}N_2O$	$[M + K-2H]^-$	354.20789	1.37	['Hexocyclium']
354.20838	5.46E+04	$C_{22}H_{29}NO_3$	$[M-H]^-$	354.20747	2.58	['Spirasine I']
355.19644	1.73E+05	$C_{14}H_{30}N_4O_5$	$[M + Na-2H]^-$	355.19629	0.42	['Fortimicin AP']
356.19983	2.90E+04	0				0
356.96521	3.79E+05	0				0
357.10168	2.01E+04	$C_{17}H_{18}N_4O_3S$	$[M-H]^-$	357.10269	−2.82	['NA', 'NA']
357.10168	2.01E+04	$C_{19}H_{18}N_2O_3$	$[M + Cl]^-$	357.10114	1.5	['NA']
357.10168	2.01E+04	$C_{20}H_{20}N_2O_2$	$[M + K-2H]^-$	357.10108	1.67	['NA']
363.01836	2.17E+04	$C_{18}H_{15}O_4P$	$[M + K-2H]^-$	363.01940	−2.88	['Triphenyl phosphate']
363.01836	2.17E+04	$C_9H_{15}N_2O_9P$	$[M + (^{37}Cl)]^-$	363.01797	1.07	['2,4-Dioxotetrahydropyrimidine *D*-ribonucleotide']
365.23699	5.55E+05	0				0
366.24036	1.13E+05	$C_{22}H_{35}NO_2$	$[M + Na-2H]^-$	366.24145	−2.97	['Himbacine']
367.13314	7.66E+04	0				0
381.23191	2.10E+06	$C_{18}H_{40}O_4P_2$	$[M-H]^-$	381.23291	−2.62	['NA']
383.22769	9.86E+04	0				0
383.23572	2.02E+04	$C_{22}H_{36}O_3$	$[M + Cl]^-$	383.23585	−0.33	['17beta-Hydroxy-2alpha-(methoxymethyl)-17-methyl-5alpha-androstan-3-one', 'Anacardic acid']
383.23572	2.02E+04	$C_{23}H_{38}O_2$	$[M + K-2H]^-$	383.23579	−0.17	['5-(Heptadec-12-enyl)resorcinol']

383.23572	2.02E+04	$C_{26}H_{34}O$	[M + Na-2H]⁻	383.23563	0.23	[‘3-(2,4-Cyclopentadien-1-ylidene)pregn-4-en-20-one’]
384.23112	2.11E+04	$C_{22}H_{37}NO_2$	[M + K-2H]⁻	384.23104	0.22	[‘Anandamide’]
384.23112	2.11E+04	$C_{25}H_{33}NO$	[M + Na-2H]⁻	384.23088	0.62	[“1’H-5alpha-Androst-2-eno[3,2-b]indol-17beta-ol”, ‘Aurachin D’]
389.19746	4.30E+04	$C_{20}H_{26}O_4$	[M + HAc-H]⁻	389.19696	1.28	[‘Carnosol’, ‘Dicyclohexyl phthalate’, ‘Gibberellin A15’]
389.19746	4.30E+04	$C_{22}H_{30}O_6$	[M-H]⁻	389.19696	1.28	[‘Laserolide’, ‘Megaphone’, ‘Neoquassin’, ‘Picrasin D’]
389.19746	4.30E+04	$C_{23}H_{30}NO_3$	[M + Na-2H]⁻	389.19724	0.57	[‘Propantheline’]
392.13566	2.11E+04	0				0
392.87632	5.51E+04	0				0
397.22685	2.93E+06	0				0
398.23021	5.60E+05	$C_{22}H_{35}NO_4$	[M + Na-2H]⁻	398.23128	−2.68	[‘Karakoline’]
399.09293	2.46E+04	$C_{13}H_{22}N_4O_8$	[M + K-2H]⁻	399.09237	1.4	[‘Clavamycin B’, ‘Clavamycin C’]
399.09293	2.46E+04	$C_{15}H_{16}O_9$	[M + HAc-H]⁻	399.09329	−0.9	[‘Cichoriin’, ‘Daphnin’, ‘Esculin’, ‘Sinapoyl malate’]
399.09293	2.46E+04	$C_{22}H_{16}N_4O_2S$	[M-H]⁻	399.09212	2.03	[‘NA’]
399.22265	1.30E+05	0				0
399.23409	3.71E+04	0				0
400.22607	2.64E+04	0				0
405.15605	6.58E+04	$C_{19}H_{22}O_6$	[M + HAc-H]⁻	405.15549	1.37	[‘Alectrol’, ‘Antheridic acid’, ‘Cynaropicrin’, ‘Gibberellin A29-catabolite’, ‘Gibberellin A3’, ‘Gibberellin A34-catabolite’, ‘Gibberellin A6’, ‘Hallactone A’, ‘Molephantin’, ‘Ponalactone A’, ‘Saupirin’, ‘Strigol’]
409.26325	5.60E+05	0				0
410.19056	1.62E+04	$C_{23}H_{29}N_3O_2S$	[M-H]⁻	410.19077	−0.52	[‘Acetophenazine’]
410.26657	1.26E+05	0				0
411.15938	1.00E+05	$C_{17}H_{24}N_2O_4S$	[M + HAc-H]⁻	411.15953	−0.37	[‘Mercaptoacetyl-Phe-Leu’]
411.20284	1.17E+05	0				0
411.25904	2.71E+04	0				0
425.25816	1.64E+06	0				0
426.26151	3.68E+05	$C_{24}H_{39}NO_4$	[M + Na-2H]⁻	426.26258	−2.5	[‘Cassaine’]
427.12425	1.42E+04	$C_{17}H_{20}O_9$	[M + HAc-H]⁻	427.12459	−0.79	[‘*O*-Feruloylquinate’]
427.19786	3.37E+04	0				0

427.25401	7.83E+04	0				0
427.26113	1.50E+04	$C_{24}H_{40}O_4$	[M + Cl]⁻	427.26206	−2.18	['12-Epideoxycholic acid', '3alpha,12alpha-Dihydroxy-5beta-cholanate', 'Allochenodeoxycholate', 'Allodeoxycholate', 'Chenodeoxycholate', 'Isochenodeoxycholate', 'Isodeoxycholate', 'Isoursodeoxycholate', 'Murideoxycholic acid', 'Ursodiol']
427.26558	2.62E+04	0				0
433.18380	2.49E+04	0				0
435.16630	5.26E+04	$C_{20}H_{24}O_7$	[M + HAc-H]⁻	435.16606	0.55	['Ailanthone', 'Euparotin', 'Eupatundin', 'Tripdiolide']
435.16630	5.26E+04	$C_{21}H_{20}N_4O_3$	[M + HAc-H]⁻	435.16739	−2.52	['NA', 'NA']
436.23260	4.76E+03	0				0
437.09170	1.10E+04	$C_{23}H_{18}N_2O_5$	[M + Cl]⁻	437.09097	1.66	['Saphenamycin']
437.21058	4.55E+03	$C_{24}H_{34}O_5$	[M + Cl]⁻	437.21003	1.27	['Dehydrocholic acid', 'Gamabufogenin', 'Telocinobufagin']
437.21058	4.55E+03	$C_{25}H_{36}O_4$	[M + K-2H]⁻	437.20997	1.4	['16alpha,17-Isopropylidenedioxy-6alpha-methylprogesterone', 'Ophiobolin A']
439.14371	4.65E+04	$C_{19}H_{24}O_6S$	[M + HAc-H]⁻	439.14322	1.13	['2-Methoxyestrone 3-sulfate']
439.14371	4.65E+04	$C_{24}H_{24}N_2O_4$	[M + Cl]⁻	439.14301	1.59	['NA', 'NA']
439.19071	6.18E+04	0				0
440.21177	1.07E+04	0				0
443.11914	2.16E+04	0				0
443.17512	3.48E+04	0				0
443.24884	1.01E+05	0				0
448.83786	2.38E+04	0				0
449.13654	1.73E+04	$C_{23}H_{26}O_7$	[M + Cl]⁻	449.13726	−1.6	['Neoisostegane']
450.83491	3.14E+04	0				0
451.16878	4.11E+04	0				0
452.17199	1.25E+04	0				0
452.83189	1.64E+04	0				0
452.88468	6.67E+03	0				0
453.28413	3.94E+04	0				0
453.28944	5.07E+05	0				0

454.14333	1.67E+04	0				0
454.29274	1.26E+05	$C_{26}H_{43}NO_4$	[M + Na-2H]⁻	454.29388	−2.5	[‘Glycolithocholate’]
455.18541	1.16E+05	$C_{24}H_{34}O_4S$	[M + (37Cl)]⁻	455.18423	2.58	[‘17beta-Hydroxy-4-mercaptoandrost-4-en-3-one 4-acetate 17-propionate’]
455.18541	1.16E+05	$C_{24}H_{34}O_6$	[M + K-2H]⁻	455.18415	2.78	[‘11beta,17,21-Trihydroxy-2alpha-methylpregn-4-ene-3,20-dione 21-acetate’, ‘21-Acetoxy-11beta,17-dihydroxy-6alpha-methylpregn-4-ene-3,20-dione’, ‘Phyllanthin’]
455.18541	1.16E+05	$C_{25}H_{30}N_4O_2$	[M + K-2H]⁻	455.18548	−0.16	[‘Naphthyl dipeptide’]
455.28518	2.80E+04	0				0
455.29169	3.85E+03	0				0
455.29679	1.76E+04	0				0
456.18866	2.84E+04	0				0
461.18213	6.30E+04	$C_{21}H_{33}N_2O_6P$	[M + Na-2H]⁻	461.18229	−0.36	[‘NA’]
461.18213	6.30E+04	$C_{28}H_{28}N_2O_2$	[M + (37Cl)]⁻	461.18153	1.3	[‘Difenoxin’]
461.21847	6.61E+04	$C_{23}H_{30}O_6$	[M + HAc-H]⁻	461.21809	0.82	[‘Citreoviridin’, ‘Cortisone acetate’, ‘NA’, ‘Prednisolone acetate’]
461.21847	6.61E+04	$C_{25}H_{34}O_8$	[M-H]⁻	461.21809	0.82	[‘Hydrocortisone succinate’]
462.17753	2.84E+04	0				0
462.22161	1.86E+04	0				0
463.23486	1.39E+05	$C_{23}H_{32}O_6$	[M + HAc-H]⁻	463.23374	2.41	[‘Cortisol 21-acetate’]
463.23486	1.39E+05	$C_{25}H_{36}O_8$	[M-H]⁻	463.23374	2.41	[‘Testosterone glucuronide’]
464.20075	1.81E+04	0				0
464.23799	3.28E+04	0				0
467.16359	6.37E+04	$C_{25}H_{28}N_2O_5S$	[M-H]⁻	467.16462	−2.2	[‘NA’]
467.16359	6.37E+04	$C_{27}H_{28}O_5$	[M + Cl]⁻	467.16308	1.1	[‘Aspulvinone H’]
468.16683	1.64E+04	0				0
469.28435	1.78E+06	0				0
470.28768	4.24E+05	$C_{26}H_{43}NO_5$	[M + Na-2H]⁻	470.28879	−2.37	[‘3alpha,12alpha-Dihydroxy-5beta-cholan-24-oylglycine’, ‘Glycochenodeoxycholate’, ‘Glycodeoxycholate’]
471.28015	8.47E+04	0				0
471.28672	1.55E+04	0				0

471.29184	3.12E+04	0				0
472.28354	2.07E+04	0				0
472.88239	1.00E+05	0				0
474.92035	2.21E+04	0				0
475.32784	4.22E+04	0				0
476.87636	3.06E+04	0				0
477.24971	1.80E+05	$C_{24}H_{34}O_6$	[M + HAc-H]$^-$	477.24939	0.66	[‘11beta,17,21-Trihydroxy-2alpha-methylpregn-4-ene-3,20-dione 21-acetate’, ‘21-Acetoxy-11beta,17-dihydroxy-6alpha-methylpregn-4-ene-3,20-dione’, ‘Phyllanthin’]
477.24971	1.80E+05	$C_{25}H_{30}N_4O_2$	[M + HAc-H]$^-$	477.25073	−2.14	[‘Naphthyl dipeptide’]
477.85383	1.02E+04	0				0
478.25300	5.15E+04	0				0
480.23300	4.68E+04	0				0
480.27921	2.64E+04	0				0
480.89629	4.55E+04	0				0
481.27978	1.66E+04	$C_{26}H_{42}O_8$	[M-H]$^-$	481.28069	−1.9	[‘Fusicoccin H’]
482.20429	6.81E+04	0				0
482.28356	1.15E+04	0				0
484.23811	1.62E+04	0				0
485.27931	2.01E+06	0				0
486.28263	4.65E+05	$C_{26}H_{43}NO_6$	[M + Na-2H]$^-$	486.28371	−2.22	[‘Glycocholate’]
487.14540	3.22E+04	0				0
487.23386	5.36E+04	$C_{23}H_{38}N_4O_3S$	[M + (^{37}Cl)]$^-$	487.23291	1.94	[‘NA’]
487.23386	5.36E+04	$C_{27}H_{36}O_8$	[M-H]$^-$	487.23374	0.24	[‘NA’]
487.27523	9.19E+04	0				0
487.28173	2.15E+04	$C_{27}H_{46}O_5$	[M + K-2H]$^-$	487.28313	−2.88	[‘3alpha,7alpha,12alpha-Trihydroxy-5beta-cholestanoate’]
487.28664	3.22E+04	$C_{27}H_{46}O_4S$	[M + Na-2H]$^-$	487.28635	0.59	[‘Cholesterol sulfate’]
488.27853	2.41E+04	$C_{25}H_{43}NO_6$	[M + Cl]$^-$	488.27844	0.18	[‘YC-17’]
488.27853	2.41E+04	$C_{29}H_{41}NO_4$	[M + Na-2H]$^-$	488.27823	0.62	[‘Buprenorphine’]
489.36458	1.66E+04	0				0
491.32308	1.61E+04	0				0

493.16236	3.36E+04	$C_{26}H_{32}O_7$	$[M + K\text{-}2H]^-$	493.16341	−2.13	['Kuraridinol', 'Kurarinol']
493.20840	5.34E+04	$C_{20}H_{42}O_7P_2$	$[M + (^{37}Cl)]^-$	493.20703	2.77	['Phytyl diphosphate']
493.20840	5.34E+04	$C_{21}H_{36}N_4O_5S$	$[M + (^{37}Cl)]^-$	493.20709	2.65	['D1927']
493.20840	5.34E+04	$C_{25}H_{28}N_8O_2$	$[M + Na\text{-}2H]^-$	493.20819	0.42	['NA']
493.20840	5.34E+04	$C_{25}H_{34}O_{10}$	$[M\text{-}H]^-$	493.20792	0.97	['Glaucarubinone', 'Soularubinone']
493.24475	9.41E+04	$C_{17}H_{34}N_6O_5S$	$[M + HAc\text{-}H]^-$	493.24499	−0.5	['Glutathionylspermidine']
493.24475	9.41E+04	$C_{24}H_{34}O_7$	$[M + HAc\text{-}H]^-$	493.24431	0.89	['Ajugarin I', 'Clerodin', 'Nigakilactone C', 'Phorbol 13-butanoate']
493.24475	9.41E+04	$C_{27}H_{34}N_4O_5$	$[M\text{-}H]^-$	493.24564	−1.81	['NA']
494.25847	1.55E+05	$C_{29}H_{37}N_3O_2$	$[M + Cl]^-$	494.25798	0.99	['Deoxytubulosine']
495.27917	1.20E+04	0				0
496.88066	2.17E+04	0				0
497.29498	1.91E+04	0				0
497.31567	3.67E+05	0				0
498.31896	9.47E+04	$C_{24}H_{49}NO_7$	$[M + Cl]^-$	498.32031	−2.7	['D-Glucosyldihydrosphingosine']
498.89563	5.52E+04	0				0
498.92405	1.12E+05	$C_{15}H_{10}O_{13}S_2$	$[M + (^{37}Cl)]^-$	498.92274	2.62	['Quercetin 3,3-bissulfate', 'Quercetin 3,4-bissulfate']
499.01785	1.21E+04	0				0
499.06046	1.53E+04	0				0
499.31112	2.61E+04	0				0
500.21500	2.23E+04	0				0
507.22397	3.20E+04	$C_{24}H_{32}O_8$	$[M + HAc\text{-}H]^-$	507.22357	0.78	['17beta-Estradiol 17-(beta-D-glucuronide)', 'Estradiol-17alpha 3-D-glucuronoside', 'Estradiol-17beta 3-glucuronide', 'Jodrellin A']
507.26111	7.89E+04	$C_{18}H_{36}N_6O_5S$	$[M + HAc\text{-}H]^-$	507.26064	0.92	['Glutathionylaminopropylcadaverine']
507.29669	6.59E+03	$C_{26}H_{40}O_6$	$[M + HAc\text{-}H]^-$	507.29634	0.68	['16-Feruloyloxypalmitate']
508.26413	1.84E+04	0				0
510.23572	3.38E+04	$C_{23}H_{33}NO_8$	$[M + HAc\text{-}H]^-$	510.23447	2.44	['NA']
510.25352	6.25E+04	$C_{29}H_{37}N_3O_3$	$[M + Cl]^-$	510.25289	1.23	['Alangimarckine', 'Tubulosine']
510.79076	9.18E+03	0				0
511.18987	4.78E+04	$C_{29}H_{32}O_6$	$[M + Cl]^-$	511.18929	1.13	['NA']

511.26751	4.72E+03	0				0
511.27410	5.62E+04	0				0
512.26915	3.60E+05	$C_{21}H_{41}N_5O_8$	$[M + Na-2H]^-$	512.27018	−2.02	['N2-Acetylgentamicin C1a']
513.27257	1.01E+05	0				0
513.28964	2.22E+05	0				0
513.31065	9.60E+05	0				0
514.28473	4.85E+06	$C_{26}H_{45}NO_7S$	$[M-H]^-$	514.28440	0.64	['Taurocholate']
514.31400	2.52E+05	0				0
515.19275	3.14E+04	$C_{14}H_{33}N_{10}O_7PS$	$[M-H]^-$	515.19193	1.59	['Phaseolotoxin']
515.19275	3.14E+04	$C_{27}H_{32}O_{10}$	$[M-H]^-$	515.19227	0.92	['Harrisonin', 'Spicatin']
515.28812	1.37E+06	0				0
515.39894	2.82E+04	0				0
515.95762	2.50E+04	0				0
516.28061	2.38E+05	$C_{25}H_{43}NO_{10}$	$[M-H]^-$	516.28142	−1.57	['Mycalamide B']
516.40226	9.37E+03	0				0
516.93090	3.79E+05	0				0
517.28435	4.86E+04	0				0
520.22672	1.71E+04	0				0
521.27581	2.76E+04	$C_{26}H_{38}O_7$	$[M + HAc-H]^-$	521.27561	0.39	['10-Desacetyltaxuyunnanin C']
525.10189	2.41E+04	0				0
526.23056	4.69E+04	0				0
526.82106	3.09E+04	0				0
527.19268	2.04E+04	$C_{24}H_{34}N_4O_5S$	$[M + (^{37}Cl)]^-$	527.19144	2.34	['Glimepiride']
528.26419	4.84E+04	$C_{26}H_{43}NO_8S$	$[M-H]^-$	528.26366	0.99	['Glycochenodeoxycholate 7-sulfate']
529.30554	1.56E+06	0				0
529.41469	2.15E+04	0				0
530.05944	1.45E+05	$C_{15}H_{17}N_7O_5S_3$	$[M + HAc-H]^-$	530.05919	0.48	['Cefmetazole']
530.05944	1.45E+05	$C_{15}H_{23}N_3O_{14}P_2$	$[M-H]^-$	530.05826	2.23	['CDP-4-dehydro-3,6-dideoxy-D-glucose', 'CDP-4-dehydro-3,6-dideoxy-D-glucose epimer']
530.05944	1.45E+05	$C_{16}H_{31}NO_{10}S_3$	$[M + K-2H]^-$	530.05962	−0.34	['Glucohirsutin']
530.30884	4.05E+05	$C_{28}H_{47}NO_7$	$[M + Na-2H]^-$	530.30992	−2.04	['Narbomycin']

531.06254	2.08E+04	0				0
531.30009	6.32E+05	$C_{27}H_{48}O_8S$	[M-H]⁻	531.29972	0.7	[‘5beta-Cyprinolsulfate’]
532.83806	4.63E+04	0				0
533.20276	5.67E+04	$C_{25}H_{30}O_9$	[M + HAc-H]⁻	533.20284	−0.15	[‘Robustaol A’]
533.20276	5.67E+04	$C_{27}H_{34}O_{11}$	[M-H]⁻	533.20284	−0.15	[‘Arctiin’, ‘Forsythin’, ‘Undulatone’]
533.29612	3.25E+04	0				0
534.83518	2.30E+04	0				0
535.29210	4.76E+04	$C_{27}H_{40}O_7$	[M + HAc-H]⁻	535.29126	1.57	[‘Cyclic-3,20-bis(1,2-ethanediyl acetal)-11alpha-(acetyloxy)-5alpha,6alpha-epoxypregnane-3,20-dione’]
535.29210	4.76E+04	$C_{29}H_{44}O_9$	[M-H]⁻	535.29126	1.57	[‘NA’, ‘Coroglaucigenin-3-*O*-alpha-L-rhamnopyranoside’, ‘Mallogenin-3-*O*-alpha-L-rhamnopyranoside’, ‘Rhodexin A’]
536.30542	5.22E+04	0				0
538.32115	5.79E+04	0				0
539.05471	1.22E+04	$C_{30}H_{16}O_8$	[M + Cl]⁻	539.05392	1.46	[‘Hypericin’]
539.22108	5.02E+04	0				0
539.25002	4.15E+04	0				0
539.44326	1.19E+04	0				0
540.33665	5.12E+04	0				0
540.93883	3.34E+05	0				0
541.34185	2.89E+05	0				0
541.41459	2.48E+04	0				0
541.94221	3.45E+04	0				0
542.34529	7.72E+04	0				0
542.41771	1.50E+04	0				0
543.23783	6.77E+04	$C_{29}H_{38}N_4O_4$	[M + K-2H]⁻	543.23791	−0.15	[‘Mucronine A’]
543.23783	6.77E+04	$C_{33}H_{36}O_7$	[M-H]⁻	543.23883	−1.84	[‘Morellin’]
543.43012	1.79E+05	0				0
544.43341	6.05E+04	0				0
545.42720	5.94E+04	0				0
547.29567	5.61E+04	$C_{27}H_{48}O_9S$	[M-H]⁻	547.29463	1.9	[‘5beta-Scymnol sulfate’]

547.29567	5.61E+04	$C_{27}H_{50}O_7P_2$	[M-H]⁻	547.29590	−0.43	['NA']
548.30799	3.37E+04	0				0
549.29177	2.16E+04	0				0
551.32241	9.32E+03	$C_{31}H_{44}N_4O_5$	[M-H]⁻	551.32389	−2.69	['Pandamine']
552.26852	8.79E+03	$C_{26}H_{46}NO_7P$	[M + (^{37}Cl)]⁻	552.26764	1.59	['2-Aminoethylphosphocholate']
552.29111	2.67E+04	$C_{30}H_{47}NO_4S$	[M + Cl]⁻	552.29198	−1.58	['NA']
552.30038	4.99E+04	0				0
552.86246	2.72E+04	0				0
554.26172	3.42E+04	0				0
554.88543	1.03E+05	0				0
556.33158	4.96E+04	0				0
556.88253	9.71E+04	0				0
557.33679	8.86E+05	0				0
557.34576	1.52E+04	0				0
558.34018	2.36E+05	0				0
558.87967	3.17E+04	0				0
559.34348	3.28E+04	0				0
560.43588	1.79E+04	0				0
563.09942	2.40E+04	$C_{30}H_{16}O_8$	[M + HAc-H]⁻	563.09837	1.86	['Hypericin']
563.32302	1.31E+04	0				0
564.28975	1.52E+04	0				0
565.22029	3.65E+04	$C_{29}H_{38}O_9$	[M + Cl]⁻	565.22099	−1.23	['Uscharidin']
565.22029	3.65E+04	$C_{30}H_{40}O_8$	[M + K-2H]⁻	565.22093	−1.13	['Terpenoid EA-I']
565.22029	3.65E+04	$C_{33}H_{36}O_7$	[M + Na-2H]⁻	565.22077	−0.86	['Morellin']
565.32337	1.97E+04	$C_{40}H_{48}$	[M + K-2H]⁻	565.32421	−1.48	['Isorenieratene']
567.28220	9.43E+04	$C_{29}H_{44}O_{11}$	[M-H]⁻	567.28109	1.96	['Sarmentoloside']
568.28570	2.44E+04	0				0
570.22763	3.02E+04	0				0
570.25680	2.83E+04	0				0
571.09753	4.87E+04	0				0

571.26913	3.53E+04	$C_{29}H_{44}O_9$	$[M + Cl]^-$	571.26794	2.09	['NA', 'Coroglaucigenin-3-*O*-alpha-L-rhamnopyranoside', 'Mallogenin-3-*O*-alpha-L-rhamnopyranoside', 'Rhodexin A']
571.26913	3.53E+04	$C_{30}H_{46}O_8$	$[M + K\text{-}2H]^-$	571.26788	2.19	['Cucurbitacin H', 'Divaricoside', 'Divostroside', 'Neriifolin']
571.26913	3.53E+04	$C_{31}H_{42}N_4O_4$	$[M + K\text{-}2H]^-$	571.26921	−0.14	['Integerrenine']
572.24338	4.18E+05	0				0
572.27241	1.06E+04	$C_{26}H_{35}N_5O_6$	$[M + HAc\text{-}H]^-$	572.27259	−0.31	['DAMGO']
572.27241	1.06E+04	$C_{28}H_{39}N_5O_8$	$[M\text{-}H]^-$	572.27259	−0.31	['Z-Gly-Pro-Leu-Gly-Pro']
573.24678	1.09E+05	$C_{29}H_{44}O_9$	$[M + K\text{-}2H]^-$	573.24714	−0.63	['NA', 'Coroglaucigenin-3-*O*-alpha-L-rhamnopyranoside', 'Mallogenin-3-*O*-alpha-L-rhamnopyranoside', 'Rhodexin A']
573.24678	1.09E+05	$C_{33}H_{36}N_4O_3$	$[M + (^{37}Cl)]^-$	573.24519	2.77	['NA']
573.24678	1.09E+05	$C_{33}H_{36}N_4O_4$	$[M + Na\text{-}2H]^-$	573.24832	−2.69	['Canthiumine']
573.33173	1.09E+06	0				0
573.34112	1.83E+04	0				0
573.42002	1.45E+04	0				0
573.91635	1.90E+04	0				0
574.24034	1.41E+05	0				0
574.33513	2.90E+05	0				0
574.88949	5.86E+04	0				0
575.28657	4.68E+04	$C_{29}H_{40}O_8$	$[M + HAc\text{-}H]^-$	575.28617	0.69	['Ajugalactone']
576.43891	2.91E+04	0				0
577.37498	1.39E+04	$C_{33}H_{54}O_8$	$[M\text{-}H]^-$	577.37459	0.67	['Asparagoside A', 'Timosaponin A-I']
577.37498	1.39E+04	$C_{37}H_{52}N_2O$	$[M + (^{37}Cl)]^-$	577.37441	0.98	['Dihydromethanophenazine']
579.86119	1.68E+04	0				0
582.27224	5.30E+04	$C_{33}H_{37}N_5O_5$	$[M\text{-}H]^-$	582.27219	0.08	['Dihydroergotamine']
584.77981	1.31E+04	0				0
584.98629	1.49E+05	0				0
585.36826	1.38E+05	0				0
585.98978	2.23E+04	0				0
586.45153	6.09E+04	0				0

587.14394	3.64E+04	0				0
587.45489	2.13E+04	0				0
588.26753	1.17E+04	0				0
592.35735	8.02E+03	0				0
593.36973	2.01E+04	0				0
594.27218	4.75E+04	0				0
595.29253	3.26E+04	$C_{33}H_{36}N_4O_3$	[M + HAc-H]⁻	595.29259	−0.11	['NA']
595.29253	3.26E+04	$C_{34}H_{44}O_9$	[M-H]⁻	595.29126	2.14	['Salannin']
595.31351	4.74E+04	$C_{29}H_{44}O_9$	[M + HAc-H]⁻	595.31239	1.88	['NA', 'Coroglaucigenin-3-*O*-alpha-L-rhamnopyranoside', 'Mallogenin-3-*O*-alpha-L-rhamnopyranoside', 'Rhodexin A']
595.38537	1.11E+04	0				0
596.28789	7.25E+05	0				0
597.29141	2.18E+05	0				0
598.42466	2.66E+04	0				0
599.24246	5.42E+04	0				0
599.26237	1.05E+05	$C_{30}H_{44}O_{10}$	[M + Cl]⁻	599.26285	−0.8	['Musaroside', 'Vernadigin']
599.49299	2.50E+04	0				0
601.36310	5.06E+05	$C_{40}H_{52}O_2$	[M + (^{37}Cl)]⁻	601.36318	−0.14	['Canthaxanthin']
601.37347	8.61E+03	0				0
602.44632	8.57E+04	0				0
602.87645	1.89E+04	0				0
603.44978	3.10E+04	0				0
604.46176	2.40E+04	0				0
606.82722	2.89E+04	0				0
607.34946	2.44E+04	0				0
607.38568	1.41E+04	0				0
608.82422	3.89E+04	0				0
609.36490	2.09E+04	0				0
610.82120	1.88E+04	0				0
612.28238	2.99E+04	0				0

614.30662	1.78E+04	0				0
614.42071	1.77E+04	0				0
617.35797	7.91E+05	$C_{40}H_{52}O_3$	[M + (^{37}Cl)]$^-$	617.35810	−0.21	[‘Phoenicoxanthin’]
617.36866	1.56E+04	0				0
618.36142	2.37E+05	0				0
618.47769	1.32E+04	0				0
618.79124	1.76E+04	0				0
619.38489	2.38E+04	0				0
621.40085	1.39E+04	0				0
623.38049	8.89E+03	0				0
629.39444	1.15E+05	0				0
630.20207	6.24E+04	0				0
630.87145	6.32E+04	0				0
631.16970	2.89E+04	$C_{34}H_{30}N_2O_9$	[M + Na-2H]$^-$	631.16980	−0.16	[‘Atalanine’]
631.29012	3.13E+04	$C_{32}H_{50}O_{10}$	[M + K-2H]$^-$	631.28901	1.76	[‘13-Deoxytedanolide’]
631.29012	3.13E+04	$C_{33}H_{46}N_4O_6$	[M + K-2H]$^-$	631.29034	−0.35	[‘L-Urobilin’]
632.86841	5.97E+04	0				0
634.86546	1.87E+04	0				0
637.39625	1.71E+04	$C_{33}H_{54}O_8$	[M + HAc-H]$^-$	637.39572	0.83	[‘Asparagoside A’, ‘Timosaponin A-I’]
642.38624	9.01E+03	0				0
643.48236	1.36E+04	0				0
644.48561	5.38E+03	0				0
649.39716	1.53E+04	$C_{34}H_{54}O_8$	[M + HAc-H]$^-$	649.39572	2.21	[‘NA’]
654.24665	1.29E+05	$C_{28}H_{39}N_7O_9$	[M + (^{37}Cl)]$^-$	654.24738	−1.12	[‘NA’]
655.19173	2.15E+05	0				0
655.33517	3.61E+04	0				0
656.19526	5.06E+04	0				0
656.24330	4.64E+04	0				0
661.38428	4.77E+05	$C_{42}H_{56}O_4$	[M + (^{37}Cl)]$^-$	661.38431	−0.05	[‘2,2-Diketospirilloxanthin’]
662.38773	1.56E+05	0				0
665.10651	5.34E+05	0				0

666.10994	1.13E+05	0				0
671.45943	6.47E+04	0				0
672.46198	2.76E+04	0				0
673.35824	6.35E+04	$C_{37}H_{54}O_{11}$	$[M-H]^-$	673.35934	−1.63	['Cimicifugoside']
673.42077	7.08E+04	0				0
674.42446	2.58E+04	0				0
675.92488	3.50E+04	0				0
676.33762	1.35E+05	0				0
677.34145	4.39E+04	0				0
677.42522	3.60E+04	0				0
678.29102	3.02E+05	0				0
679.29484	9.40E+04	0				0
683.36622	2.07E+04	0				0
691.42164	2.42E+04	0				0
691.44084	3.85E+04	0				0
692.44440	1.23E+04	0				0
692.82422	1.78E+04	0				0
694.14112	9.39E+04	0				0
694.25609	8.53E+04	$C_{25}H_{47}N_5O_{15}$	$[M + K-2H]^-$	694.25548	0.88	["2″-*N*-Acetyl-6″-deamino-6″-hydroxyneomycin C″]
697.13121	7.01E+04	0				0
698.50394	1.81E+04	0				0
699.32254	1.85E+04	0				0
701.46994	3.66E+04	0				0
706.41393	1.31E+05	0				0
709.13264	3.52E+05	0				0
710.13596	7.23E+04	0				0
737.12695	1.94E+05	0				0
741.15799	3.28E+05	0				0
742.14956	2.53E+04	$C_{21}H_{31}N_7O_{15}P_2$	$[M + HAc-H]^-$	742.14920	0.49	['(6S)-6-Hydroxy-1,4,5,6-tetrahydronicotinamide-adenine dinucleotide']
749.43696	2.11E+05	$C_{46}H_{64}O_6$	$[M + (^{37}Cl)]^-$	749.43674	0.29	['Hydroxychlorobactene glucoside']

750.44030	7.83E+04	0				0
751.17866	8.18E+04	0				0
753.12156	2.01E+05	0				0
753.15872	3.95E+05	0				0
754.27723	1.54E+05	0				0
756.17935	3.15E+04	0				0
760.29424	1.13E+05	0				0
771.13241	1.68E+05	0				0
772.13675	4.43E+04	0				0
775.95465	7.47E+04	0				0
776.95190	1.60E+06	0				0
781.19021	1.37E+05	0				0
785.11885	4.88E+04	0				0

Table S2. Putative annotations of metabolites measured in the SDB-RPS trout class using FT-ICR mass spectrometry.

m/z	Median Intensity	Empirical Formula	Ion Form	Theoretical Mass (Da)	Mass Error (ppm)	KEGG_COMPOUND
103.97648	8.56E+03	0				0
121.94437	2.56E+03	0				0
122.97568	3.20E+03	$C_2H_4O_4S$	$[M-H]^-$	122.97576	−0.61	['Sulfoacetaldehyde']
122.97568	3.20E+03	O_2S	$[M + HAc-H]^-$	122.97576	−0.61	['Sulfur dioxide']
130.88913	7.42E+02	0				0
132.94633	2.07E+03	H_3O_4P	$[M + Cl]^-$	132.94630	0.23	['Orthophosphate']
134.00740	5.46E+02	0				0
136.96607	9.83E+02	0				0
136.97317	4.42E+03	0				0
138.97059	2.38E+03	$C_2H_4O_5S$	$[M-H]^-$	138.97067	−0.58	['Sulfoacetate']
144.86961	4.45E+03	0				0
146.86666	5.29E+03	0				0
148.86371	1.93E+03	0				0
152.93651	2.98E+03	0				0

153.92246	2.41E+03	0				0
154.89841	1.08E+03	0				0
168.91422	1.81E+04	0				0
168.98944	2.22E+04	0				0
170.91127	1.32E+04	0				0
171.88873	3.88E+03	0				0
172.07685	3.58E+03	$C_{11}H_{11}NO$	$[M-H]^-$	172.07679	0.36	['1,3-Dimethyl-8-isoquinolinol', 'Pyroquilon']
174.93117	7.08E+03	0				0
176.92822	4.83E+03	0				0
176.98113	7.19E+03	0				0
177.90568	2.39E+03	0				0
178.01803	1.93E+03	$C_5H_9NO_4S$	$[M-H]^-$	178.01795	0.42	['S-Carboxymethyl-L-cysteine']
178.94304	7.84E+03	0				0
179.90274	1.66E+03	0				0
179.96275	7.21E+03	0				0
182.93727	1.89E+03	0				0
185.02787	1.16E+05	0				0
185.98774	1.91E+03	0				0
186.03123	1.16E+04	0				0
187.02369	6.55E+03	$C_6H_6N_4O_2$	$[M + Na-2H]^-$	187.02374	−0.29	['1-Methylxanthine', '3-Methylxanthine', '7-Methylxanthine']
187.03214	9.97E+02	0				0
193.03547	4.03E+03	$C_{10}H_8N_2$	$[M + (^{37}Cl)]^-$	193.03520	1.4	['3-Indoleacetonitrile']
193.03547	4.03E+03	$C_4H_6O_5$	$[M + HAc-H]^-$	193.03538	0.47	['(R)-Malate', '(S)-Malate', '3-Dehydro-L-threonate', 'Malate']
193.03547	4.03E+03	$C_6H_{10}O_7$	$[M-H]^-$	193.03538	0.47	['2-Dehydro-D-galactonate', '2-Keto-D-gluconic acid', '3-Dehydro-L-gulonate', '5-Dehydro-D-gluconate', 'D-Fructuronate', 'D-Galacturonate', 'D-Glucuronate', 'D-Glucuronic acid', 'D-Mannuronate', 'D-Tagaturonate', 'Galacturonic acid', 'L-Guluronic acid', 'L-Iduronic acid', 'beta-D-Glucopyranuronic acid']

193.05405	5.72E+02	$C_{10}H_{10}N_2$	[M + Cl]⁻	193.05380	1.3	['1,5-Naphthalenediamine', 'Nicotyrine']
193.05405	5.72E+02	$C_5H_{10}O_2S$	[M + HAc-H]⁻	193.05401	0.23	['S,S-Dimethyl-beta-propiothetin']
195.93322	4.77E+04	0				0
196.95161	6.69E+03	0				0
198.90774	1.09E+04	0				0
199.03915	6.33E+02	0				0
200.04690	4.61E+03	0				0
201.03937	2.08E+03	$C_7H_8N_4O_2$	[M + Na-2H]⁻	201.03939	−0.12	['1,7-Dimethylxanthine', 'Theobromine', 'Theophylline']
201.95018	1.31E+04	0				0
204.00015	5.63E+03	0				0
204.92470	6.19E+03	0				0
205.00099	7.37E+02	0				0
206.92175	2.19E+03	0				0
207.01398	6.67E+02	0				0
208.84540	4.89E+03	0				0
209.94916	4.45E+03	0				0
210.84244	7.15E+03	0				0
211.04029	1.31E+03	$C_{11}H_{12}NO$	[M + K-2H]⁻	211.04050	−0.97	['Echinorine']
212.83950	3.44E+03	0				0
214.01567	1.07E+03	0				0
214.51190	8.58E+02	0				0
214.83662	1.00E+03	0				0
215.34673	3.41E+03	0				0
216.97371	1.78E+03	0				0
217.06213	1.49E+03	0				0
219.97770	1.37E+05	$C_3H_8NO_6P$	[M + Cl]⁻	219.97833	−2.86	['*D*-*O*-Phosphoserine', '*O*-Phospho-L-serine']
219.97770	1.37E+05	$C_4H_{10}NO_3PS$	[M + (³⁷Cl)]⁻	219.97836	−2.98	['Acephate']
219.97958	3.16E+03	0				0
220.87126	1.81E+03	0				0
220.98105	5.76E+03	$C_4H_6N_4O_3S_2$	[M-H]⁻	220.98086	0.87	['Acetazolamide']
222.96932	4.20E+03	0				0

224.04802	2.13E+03	$C_{11}H_{11}NO_2$	$[M + Cl]^-$	224.04838	−1.61	['1,3-Dimethyl-6,8-isoquinolinediol', 'Backebergine', 'Phensuximide']
250.91738	5.22E+04	0				0
252.91445	4.28E+04	0				0
256.93435	5.45E+04	0				0
258.93139	3.60E+04	$C_6H_6O_7S$	$[M + K\text{-}2H]^-$	258.93203	−2.49	['3-Sulfomuconate', '4-Sulfolactone']
265.14806	2.40E+06	0				0
265.15105	9.49E+04	0				0
266.15142	3.04E+05	$C_{11}H_{17}N_3O$	$[M + HAc\text{-}H]^-$	266.15102	1.52	['Alchornine', 'Arenaine']
267.14388	1.01E+05	0				0
293.17941	2.25E+06	0				0
294.18277	3.94E+05	$C_{13}H_{21}N_3O$	$[M + HAc\text{-}H]^-$	294.18232	1.54	['Procainamide']
295.17519	8.82E+04	0				0
301.98083	4.37E+04	0				0
307.05022	2.91E+04	0				0
308.87608	3.02E+04	0				0
309.17434	2.31E+06	$C_{17}H_{26}N_2O$	$[M + Cl]^-$	309.17391	1.38	['Ropivacaine', 'Sauroxine', 'alpha-Obscurine']
309.17434	2.31E+06	$C_{18}H_{20}N$	$[M + HAc\text{-}H]^-$	309.17343	2.95	['cis-*N*-Methyl-(*S*)-7,8,13,14-tetrahydroprotoberberine']
310.17770	3.48E+05	0				0
310.87314	2.73E+04	0				0
313.14477	1.86E+04	$C_{19}H_{22}O_4$	$[M\text{-}H]^-$	313.14453	0.75	['2,3-Dehydro-gibberellin A9', 'Heliettin']
316.89008	4.17E+04	0				0
321.21072	6.93E+05	0				0
322.21407	1.18E+05	$C_{15}H_{25}N_3O$	$[M + HAc\text{-}H]^-$	322.21362	1.41	['Triapenthenol']
323.10677	8.07E+04	$C_{15}H_{20}N_2O_4S$	$[M\text{-}H]^-$	323.10710	−1.03	['Acetohexamide', 'Chromanol 293B', 'HMR1556']
324.92191	1.37E+04	0				0
327.97562	3.40E+04	0				0
332.92053	1.44E+05	$C_{14}H_4N_2O_2S_2$	$[M + K\text{-}2H]^-$	332.92003	1.51	['Dithianon']
333.00769	2.65E+04	0				0
335.22633	3.24E+04	0				0
337.20567	2.36E+06	0				0

337.20999	1.51E+05	0				0
338.20901	4.34E+05	0				0
338.93750	1.13E+05	0				0
340.93456	7.47E+04	0				0
340.98746	3.48E+04	$C_9H_{21}O_3PS_3$	[M + K-2H]⁻	340.98766	−0.57	['IPSP']
342.98471	1.56E+04	0				0
343.01719	2.11E+04	0				0
349.00268	1.94E+04	0				0
350.12574	7.53E+04	0				0
351.13810	7.75E+04	$C_{17}H_{24}N_2O_4S$	[M-H]⁻	351.13840	−0.86	['Mercaptoacetyl-Phe-Leu']
353.05253	6.66E+04	0				0
353.20056	3.78E+06	0				0
353.92262	5.68E+04	0				0
354.05589	1.07E+04	0				0
354.20395	6.51E+05	0				0
354.20841	4.86E+04	$C_{20}H_{33}N_2O$	[M + K-2H]⁻	354.20789	1.46	['Hexocyclium']
354.20841	4.86E+04	$C_{22}H_{29}NO_3$	[M-H]⁻	354.20747	2.66	['Spirasine I']
355.19647	1.51E+05	$C_{14}H_{30}N_4O_5$	[M + Na-2H]⁻	355.19629	0.51	['Fortimicin AP']
357.10163	1.81E+04	0				0
358.96207	1.24E+05	0				0
359.20787	2.30E+04	0				0
362.96992	7.27E+04	0				0
365.23701	5.01E+05	0				0
366.24036	1.03E+05	$C_{22}H_{35}NO_2$	[M + Na-2H]⁻	366.24145	−2.97	['Himbacine']
369.98027	1.79E+04	0				0
374.84887	1.44E+04	0				0
376.97611	1.24E+06	$C_{12}H_9N_2O_8P$	[M + (^{37}Cl)]⁻	376.97611	0.01	['Bis-4-nitrophenyl phosphate']
381.19232	2.70E+04	$C_{18}H_{26}O_5$	[M + HAc-H]⁻	381.19188	1.16	['alpha-Zearalanol', 'beta-Zearalanol']
381.19232	2.70E+04	$C_{20}H_{30}O_7$	[M-H]⁻	381.19188	1.16	['Cinncassiol A', 'Cinncassiol C3']
381.23194	1.90E+06	0				0
382.23527	3.91E+05	0				0

383.22768	8.90E+04	0				0
383.98408	3.92E+04	$C_9H_{17}NO_9S_2$	[M + K-2H]⁻	383.98308	2.59	['Glucolepidiin']
384.93100	2.92E+04	0				0
385.05203	1.51E+04	0				0
390.87923	5.44E+04	0				0
394.15192	5.24E+04	0				0
397.15866	2.28E+05	0				0
397.22686	2.60E+06	0				0
398.23022	5.07E+05	$C_{22}H_{35}NO_4$	[M + Na-2H]⁻	398.23128	−2.66	['Karakoline']
398.89324	2.91E+04	0				0
399.22262	1.14E+05	0				0
399.23411	3.37E+04	0				0
400.90805	2.41E+04	0				0
401.12484	1.89E+04	$C_{19}H_{18}O_6$	[M + HAc-H]⁻	401.12419	1.61	['3-(4-Methoxyphenyl)-5,6,7-trimethoxy-4H-1-benzopyran-4-one', "4',5,6,7-Tetramethoxyflavone", 'UWM6']
401.12484	1.89E+04	$C_{21}H_{22}O_8$	[M-H]⁻	401.12419	1.61	["(3'R,4'R)-3-Epoxyangeloyloxy-4-acetoxy-3',4-dihydroseselin", '2-(2,5-Dimethoxyphenyl)-5,6,7,8-tetramethoxy-4H-1-benzopyran-4-one', '2-(3,5-Dimethoxyphenyl)-5,6,7,8-tetramethoxy-4H-1-benzopyran-4-one', 'Flavanone 7-*O*-beta-D-glucoside', 'Nobiletin']
403.21325	4.04E+04	$C_{21}H_{28}O_4$	[M + HAc-H]⁻	403.21261	1.58	['11-Dehydrocorticosterone', '21-Deoxycortisone', '3beta-Hydroxy-17-oxoandrost-5-en-19-al acetate', 'Bisphenol A bis(2-hydroxypropyl) ether'] .
403.21325	4.04E+04	$C_{23}H_{32}O_6$	[M-H]⁻	403.21261	1.58	['Cortisol 21-acetate']
411.20281	1.02E+05	0				0
412.96680	1.50E+05	0				0
415.17680	2.61E+04	$C_{21}H_{24}O_5$	[M + HAc-H]⁻	415.17623	1.38	['Denudatin B', 'Gingerenone A', 'Kadsurenone', 'Rutamarin']
415.17680	2.61E+04	$C_{23}H_{28}O_7$	[M-H]⁻	415.17623	1.38	['Erioflorin methacrylate']
416.92080	7.66E+04	0				0
418.21690	2.64E+04	0				0

420.94069	5.17E+04	0				0
422.99070	2.31E+04	0				0
425.25819	1.48E+06	0				0
426.26154	3.29E+05	$C_{24}H_{39}NO_4$	[M + Na-2H]⁻	426.26258	−2.43	['Cassaine']
427.17681	1.83E+04	$C_{22}H_{24}O_5$	[M + HAc-H]⁻	427.17623	1.36	['4,4-Bis[4-(acetyloxy)phenyl]3-hexanone']
427.17681	1.83E+04	$C_{24}H_{28}O_7$	[M-H]⁻	427.17623	1.36	['psi-Rhodomyrtoxin']
427.25396	6.82E+04	0				0
427.26560	2.18E+04	0				0
429.08315	1.80E+04	0				0
431.20830	2.22E+04	$C_{22}H_{28}O_5$	[M + HAc-H]⁻	431.20753	1.79	['(+)-Veraguensin', 'Pyrethrin II', 'Saucernetin']
431.20830	2.22E+04	$C_{24}H_{32}O_7$	[M-H]⁻	431.20753	1.79	['Grandisin', 'Inflexin', 'Magnosalicin', 'Schizandrin']
435.93121	1.89E+05	0				0
436.16971	1.09E+04	0				0
436.23263	4.11E+03	0				0
436.23784	6.65E+04	0				0
436.92492	7.92E+05	0				0
436.93049	3.06E+04	0				0
437.92825	6.05E+04	0				0
438.25372	1.44E+04	0				0
439.14376	3.96E+04	$C_{19}H_{24}O_6S$	[M + HAc-H]⁻	439.14322	1.24	['2-Methoxyestrone 3-sulfate']
439.15364	1.19E+04	$C_{22}H_{28}O_7$	[M + Cl]⁻	439.15291	1.67	['Chromolaenide', 'Eupacunin', 'Eupaserrin', 'Eupatocunin', 'Isodonal', 'Ursiniolide A']
439.15364	1.19E+04	$C_{23}H_{30}O_6$	[M + K-2H]⁻	439.15285	1.81	['Citreoviridin', 'Cortisone acetate', 'Prednisolone acetate']
439.15364	1.19E+04	$C_{24}H_{20}N_6O_3$	[M-H]⁻	439.15241	2.79	['Candesartan']
439.96489	8.62E+04	0				0
440.14695	1.18E+04	$C_{21}H_{27}NO_7$	[M + Cl]⁻	440.14816	−2.74	['Clivoline']
441.10188	3.07E+04	$C_{17}H_{24}O_{12}$	[M + Na-2H]⁻	441.10145	0.98	['Secogalioside']
441.24824	1.67E+05	$C_{21}H_{34}O_6$	[M + HAc-H]⁻	441.24939	−2.61	['Sarcostin']
441.25308	1.92E+06	0				0
441.94274	3.56E+04	0				0
442.25642	4.28E+05	$C_{24}H_{39}NO_5$	[M + Na-2H]⁻	442.25749	−2.43	['Talatizamine']

443.13898	1.20E+04	$C_{23}H_{24}N_2O_5$	[M + Cl]⁻	443.13792	2.38	['Bleekerine']
443.13898	1.20E+04	$C_{24}H_{26}N_2O_4$	[M + K-2H]⁻	443.13786	2.52	['Carvedilol']
443.24879	9.10E+04	0				0
443.26070	2.47E+04	0				0
444.17830	8.83E+03	0				0
446.18252	1.59E+04	0				0
447.20299	4.88E+04	$C_{22}H_{28}O_6$	[M + HAc-H]⁻	447.20244	1.22	['Quassin', 'Surinamensin']
447.20299	4.88E+04	$C_{24}H_{32}O_8$	[M-H]⁻	447.20244	1.22	['17beta-Estradiol 17-(beta-D-glucuronide)', 'Estradiol-17alpha 3-D-glucuronoside', 'Estradiol-17beta 3-glucuronide', 'Jodrellin A']
447.23941	3.56E+04	$C_{18}H_{36}N_6O_5S$	[M-H]⁻	447.23951	−0.23	['Glutathionylaminopropylcadaverine']
447.23941	3.56E+04	$C_{23}H_{32}O_5$	[M + HAc-H]⁻	447.23883	1.3	['11alpha,17beta-Dihydroxyandrost-4-en-3-one diacetate', '11beta,21-Dihydroxypregn-4-ene-3,20-dione 21-acetate', '6beta,17beta-Dihydroxyandrost-4-en-3-one diacetate']
447.95970	1.01E+04	0				0
448.83786	2.04E+04	0				0
450.28993	1.32E+04	0				0
450.90169	4.11E+04	0				0
452.89876	1.56E+04	0				0
452.92610	9.48E+03	0				0
453.19247	1.30E+04	$C_{26}H_{30}O_7$	[M-H]⁻	453.19188	1.3	['Obacunone']
454.14330	1.46E+04	0				0
454.85485	1.48E+04	0				0
454.91638	2.77E+04	0				0
455.20801	2.58E+04	$C_{26}H_{32}O_7$	[M-H]⁻	455.20753	1.06	['Kuraridinol', 'Kurarinol']
455.20801	2.58E+04	$C_{30}H_{30}N_2$	[M + (³⁷Cl)]⁻	455.20735	1.45	['4,4-(Diphenylethenylidene)bis[*N*,*N*-dimethylbenzenamine]']
456.89534	8.86E+03	0				0
457.22373	3.36E+04	$C_{26}H_{34}O_7$	[M-H]⁻	457.22318	1.21	['Cinobufotalin', 'Fumagillin', 'Hellebrigenin 3-acetate']
458.84890	8.93E+03	0				0
458.90189	8.49E+03	0				0

458.97819	1.99E+05	0				0
461.94672	7.35E+06	0				0
462.24967	6.42E+03	0				0
462.26855	1.12E+04	0				0
462.95011	6.13E+05	0				0
463.19780	6.35E+04	$C_{22}H_{28}O_7$	[M + HAc-H]⁻	463.19736	0.95	[‘Chromolaenide’, ‘Eupacunin’, ‘Eupaserrin’, ‘Eupatocunin’, ‘Isodonal’, ‘Ursiniolide A’]
463.19780	6.35E+04	$C_{24}H_{32}O_9$	[M-H]⁻	463.19736	0.95	[‘16-Glucuronide-estriol’, ‘16alpha,17beta-Estriol 17-(beta-D-glucuronide)’, ‘16alpha,17beta-Estriol 3-(beta-D-glucuronide)’, ‘Renillafoulin A’]
463.95077	9.11E+04	0				0
464.27298	7.96E+03	$C_{17}H_{35}N_5O_6$	[M + HAc-H]⁻	464.27259	0.84	[‘Fortimicin A’]
465.19706	5.10E+03	$C_{24}H_{32}N_2O_5$	[M + (^{37}Cl)]⁻	465.19757	−1.11	[‘Aspidoalbine’]
465.98705	4.78E+04	0				0
466.93389	1.10E+04	0				0
467.17157	1.56E+04	$C_{25}H_{20}N_4O_2$	[M + HAc-H]⁻	467.17248	−1.95	[‘Devazepide’]
468.93154	1.38E+04	0				0
469.28437	1.61E+06	0				0
470.28770	3.84E+05	$C_{26}H_{43}NO_5$	[M + Na-2H]⁻	470.28879	−2.32	[‘3alpha,12alpha-Dihydroxy-5beta-cholan-24-oylglycine’, ‘Glycochenodeoxycholate’, ‘Glycodeoxycholate’]
470.89208	3.84E+04	0				0
471.05616	2.08E+04	0				0
471.16648	2.16E+04	$C_{25}H_{28}O_9$	[M-H]⁻	471.16606	0.89	[‘C1-C9-Glycosylated UWM6’]
471.17125	9.51E+03	0				0
471.20278	4.43E+04	$C_{21}H_{32}N_4O_6$	[M + Cl]⁻	471.20159	2.53	[‘HC-toxin’]
471.20278	4.43E+04	$C_{25}H_{24}N_4O_2$	[M + HAc-H]⁻	471.20378	−2.12	[‘GF 109203X’]
471.20278	4.43E+04	$C_{26}H_{32}O_8$	[M-H]⁻	471.20244	0.71	[‘Bryophyllin A’, ‘Deoxylimonate’]
471.20848	5.92E+03	$C_{21}H_{39}O_7P$	[M + (^{37}Cl)]⁻	471.20979	−2.79	[‘Oleoylglycerone phosphate’]
472.28356	1.88E+04	0				0
472.88241	8.97E+04	0				0

473.21861	5.77E+04	$C_{24}H_{30}O_6$	[M + HAc-H]⁻	473.21809	1.09	['Eplerenone', 'Estra-1,3,5(10)-triene-3,6alpha,17beta-triol triacetate', 'Estra-1,3,5(10)-triene-3,6beta,17beta-triol triacetate', 'Magnoshinin']
473.21861	5.77E+04	$C_{26}H_{34}O_8$	[M-H]⁻	473.21809	1.09	['Agrimophol', 'Picrasin A']
473.29115	1.68E+04	$C_{26}H_{38}O_4$	[M + HAc-H]⁻	473.29086	0.61	['Lupulone']
474.27939	8.30E+03	$C_{28}H_{41}NO_3$	[M + Cl]⁻	474.27805	2.83	['Arachidonoyl dopamine']
474.87941	8.52E+04	0				0
475.32790	3.76E+04	0				0
475.85683	1.06E+04	0				0
476.33127	1.01E+04	0				0
476.87638	2.69E+04	0				0
477.24973	1.62E+05	$C_{24}H_{34}O_6$	[M + HAc-H]⁻	477.24939	0.7	['11beta,17,21-Trihydroxy-2alpha-methylpregn-4-ene-3,20-dione 21-acetate', '21-Acetoxy-11beta,17-dihydroxy-6alpha-methylpregn-4-ene-3,20-dione', 'Phyllanthin']
477.24973	1.62E+05	$C_{25}H_{30}N_4O_2$	[M + HAc-H]⁻	477.25073	−2.09	['Naphthyl dipeptide']
478.14982	1.86E+04	$C_{24}H_{21}NO_6$	[M + HAc-H]⁻	478.15074	−1.93	['Jadomycin A']
478.25300	4.58E+04	0				0
478.89920	4.25E+04	0				0
479.22974	1.71E+05	0				0
480.27923	2.33E+04	0				0
480.89631	4.00E+04	0				0
481.20084	1.97E+04	$C_{25}H_{34}O_7$	[M + Cl]⁻	481.19986	2.04	['Clavulone I', 'Clavulone II', 'Clavulone III', 'Clavulone IV', 'Gradolide']
481.20084	1.97E+04	$C_{26}H_{36}O_6$	[M + K-2H]⁻	481.19980	2.17	['Bufotalin']
481.29979	1.39E+04	0				0
482.89344	1.23E+04	0				0
482.91869	4.06E+04	0				0
484.23812	1.47E+04	0				0
485.18214	2.54E+04	$C_{24}H_{26}O_7$	[M + HAc-H]⁻	485.18171	0.89	['Archangelicin', 'Disenecionyl cis-khellactone']
485.18214	2.54E+04	$C_{26}H_{30}O_9$	[M-H]⁻	485.18171	0.89	['Nafenopin glucuronide', 'Rutaevin']
485.27932	1.83E+06	0				0

486.28264	4.20E+05	$C_{26}H_{43}NO_6$	[M + Na-2H]⁻	486.28371	−2.2	['Glycocholate']
487.20392	3.50E+03	0				0
487.27522	8.10E+04	0				0
487.30597	1.08E+06	$C_{27}H_{40}O_4$	[M + HAc-H]⁻	487.30651	−1.12	['Hydroxyprogesterone caproate']
488.19280	1.42E+04	0				0
488.30928	2.90E+05	0				0
489.30299	3.49E+05	0				0
490.30631	9.19E+04	0				0
490.86348	2.53E+04	0				0
490.92508	8.83E+03	0				0
491.32288	1.40E+04	0				0
492.18808	1.35E+04	0				0
492.88645	1.62E+05	0				0
493.24489	8.27E+04	$C_{17}H_{34}N_6O_5S$	[M + HAc-H]⁻	493.24499	−0.21	['Glutathionylspermidine']
493.24489	8.27E+04	$C_{24}H_{34}O_7$	[M + HAc-H]⁻	493.24431	1.18	['Ajugarin I', 'Clerodin', 'Nigakilactone C', 'Phorbol 13-butanoate']
494.25848	1.38E+05	$C_{29}H_{37}N_3O_2$	[M + Cl]⁻	494.25798	1.01	['Deoxytubulosine']
494.88351	9.98E+04	0				0
494.93645	2.04E+05	0				0
495.26176	4.68E+04	0				0
495.93634	1.57E+04	0				0
496.25478	1.22E+04	$C_{29}H_{37}N_3O_2$	[M + (^{37}Cl)]⁻	496.25503	−0.5	['Deoxytubulosine']
496.27414	2.11E+05	$C_{21}H_{41}N_5O_7$	[M + Na-2H]⁻	496.27527	−2.27	['Netilmicin']
496.88056	1.87E+04	0				0
496.89823	9.51E+04	0				0
497.15948	1.41E+04	$C_{25}H_{32}O_8$	[M + K-2H]⁻	497.15833	2.32	['17,21-Dihydroxypregn-4-ene-3,11,20-trione 21-(hydrogensuccinate)', 'Aspidin', 'alpha-Kosin']
497.29493	1.72E+04	0				0
497.91098	4.45E+04	0				0
497.94432	7.48E+03	0				0
498.26993	1.14E+04	$C_{20}H_{41}N_5O_7$	[M + Cl]⁻	498.27000	−0.14	['Gentamicin C2', 'Gentamicin C2b']

499.21182	8.06E+04	$C_{25}H_{36}O_8$	$[M + Cl]^-$	499.21042	2.8	['Testosterone glucuronide']
499.21182	8.06E+04	$C_{26}H_{38}O_7$	$[M + K\text{-}2H]^-$	499.21036	2.92	['10-Desacetyltaxuyunnanin C']
501.20699	1.25E+04	$C_{25}H_{36}O_8$	$[M + (^{37}Cl)]^-$	501.20747	−0.96	['Testosterone glucuronide']
501.28513	9.55E+03	0				0
503.15607	1.04E+04	$C_{25}H_{28}O_{11}$	$[M\text{-}H]^-$	503.15589	0.36	['Sergeolide', 'Urdamycinone F']
504.96518	3.74E+04	0				0
504.99350	2.18E+04	0				0
510.23560	3.01E+04	0				0
510.25353	5.60E+04	$C_{29}H_{37}N_3O_3$	$[M + Cl]^-$	510.25289	1.25	['Alangimarckine', 'Tubulosine']
510.26142	3.19E+03	$C_{27}H_{41}NO_6$	$[M + Cl]^-$	510.26279	−2.69	['Progeldanamycin']
510.93425	7.29E+04	0				0
511.25599	3.51E+04	0				0
511.27414	4.94E+04	0				0
511.35047	6.88E+04	0				0
511.91733	1.20E+05	0				0
512.21407	8.31E+03	0				0
512.26206	1.96E+04	0				0
512.26916	3.23E+05	$C_{21}H_{41}N_5O_8$	$[M + Na\text{-}2H]^-$	512.27018	−2	['N2-Acetylgentamicin C1a']
513.27262	9.00E+04	0				0
513.28275	1.00E+04	0				0
513.28965	1.97E+05	0				0
513.31068	8.37E+05	0				0
513.88848	3.20E+05	0				0
514.28474	4.32E+06	$C_{26}H_{45}NO_7S$	$[M\text{-}H]^-$	514.28440	0.66	['Taurocholate']
514.30183	3.52E+04	0				0
514.31398	2.26E+05	0				0
514.32493	2.13E+05	0				0
514.90699	3.30E+04	0				0
515.27075	6.49E+03	$C_{30}H_{42}N_2O_2S$	$[M + Na\text{-}2H]^-$	515.27137	−1.2	['Thiobinupharidine']
515.28814	1.21E+06	0				0
515.32833	6.19E+04	0				0

515.88554	1.08E+05	0				0
516.28062	2.13E+05	$C_{25}H_{43}NO_{10}$	[M-H]⁻	516.28142	−1.55	['Mycalamide B′]
516.29185	1.39E+05	0				0
516.93091	3.68E+05	0				0
517.28457	4.05E+04	0				0
518.05949	8.44E+03	0				0
518.92801	1.14E+05	0				0
518.98088	4.14E+05	0				0
519.18728	3.56E+04	$C_{24}H_{28}O_9$	[M + HAc-H]⁻	519.18719	0.18	['6-Acetylpicropolin′]
519.18728	3.56E+04	$C_{26}H_{32}O_{11}$	[M-H]⁻	519.18719	0.18	['(-)-Pinoresinol glucoside', 'Brusatol′]
519.90545	1.95E+05	0				0
520.21941	1.80E+04	$C_{18}H_{37}N_5O_{10}$	[M + (^{37}Cl)]⁻	520.22050	−2.09	['Kanamycin B′]
520.94277	4.37E+05	0				0
521.90250	6.57E+04	0				0
521.93803	6.06E+04	0				0
521.95537	1.25E+05	0				0
522.27109	8.41E+03	0				0
523.27453	1.22E+04	0				0
523.91731	9.09E+04	0				0
526.23053	4.07E+04	0				0
526.24937	5.61E+03	0				0
527.19268	1.79E+04	$C_{24}H_{34}N_4O_5S$	[M + (^{37}Cl)]⁻	527.19144	2.34	['Glimepiride′]
528.26421	4.28E+04	$C_{26}H_{43}NO_8S$	[M-H]⁻	528.26366	1.03	['Glycochenodeoxycholate 7-sulfate′]
529.17198	1.31E+04	$C_{27}H_{30}O_{11}$	[M-H]⁻	529.17154	0.83	['Curcumin monoglucoside′]
529.28494	6.72E+04	0				0
529.30554	1.39E+06	0				0
530.05944	1.25E+05	$C_{15}H_{17}N_7O_5S_3$	[M + HAc-H]⁻	530.05919	0.48	['Cefmetazole′]
530.05944	1.25E+05	$C_{15}H_{23}N_3O_{14}P_2$	[M-H]⁻	530.05826	2.23	['CDP-4-dehydro-3,6-dideoxy-D-glucose′, 'CDP-4-dehydro-3,6-dideoxy-D-glucose epimer′]
530.05944	1.25E+05	$C_{16}H_{31}NO_{10}S_3$	[M + K-2H]⁻	530.05962	−0.34	['Glucohirsutin′]
530.23907	1.21E+04	$C_{24}H_{39}N_5O_6$	[M + K-2H]⁻	530.23864	0.81	['Syringolin A′]

530.23907	1.21E+04	$C_{28}H_{37}NO_9$	[M-H]⁻	530.23956	−0.92	[‘Harringtonine’]
530.30883	3.62E+05	$C_{28}H_{47}NO_7$	[M + Na-2H]⁻	530.30992	−2.06	[‘Narbomycin’]
530.84105	3.55E+04	0				0
531.06253	1.85E+04	0				0
531.23647	1.51E+04	0				0
531.30008	5.77E+05	$C_{27}H_{48}O_8S$	[M-H]⁻	531.29972	0.69	[‘5beta-Cyprinolsulfate’]
531.36985	7.05E+03	$C_{30}H_{48}O_4$	[M + HAc-H]⁻	531.36911	1.39	[‘Alisol B’, ‘Alphitolic acid’, ‘Crataegolic acid’, ‘Echinocystic acid’, ‘Gratiogenin’, ‘Siaresinol’, ‘Sumaresinol’]
532.21871	1.68E+04	$C_{25}H_{31}NO_8$	[M + HAc-H]⁻	532.21882	−0.21	[‘Senampeline A’]
532.30348	1.60E+05	0				0
532.83809	4.16E+04	0				0
533.29612	2.89E+04	0				0
534.29915	8.24E+03	0				0
534.83508	2.06E+04	0				0
535.18240	3.41E+04	0				0
535.29190	4.33E+04	$C_{27}H_{40}O_7$	[M + HAc-H]⁻	535.29126	1.2	[‘Cyclic-3,20-bis(1,2-ethanediyl acetal)-11alpha-(acetyloxy)-5alpha,6alpha-epoxypregnane-3,20-dione’]
535.29190	4.33E+04	$C_{29}H_{44}O_9$	[M-H]⁻	535.29126	1.2	[‘Coroglaucigenin-3-*O*-alpha-L-rhamnopyranoside’, ‘Mallogenin-3-*O*-alpha-L-rhamnopyranoside’, ‘Rhodexin A’]
536.30537	4.67E+04	0				0
536.85766	2.14E+04	0				0
537.21317	1.09E+04	0				0
537.30847	2.20E+04	$C_{30}H_{48}N_2O_4$	[M + K-2H]⁻	537.31001	−2.87	[‘Vicenistatin’]
537.93287	1.61E+06	0				0
538.32107	5.15E+04	0				0
538.85484	2.36E+04	0				0
538.93631	1.63E+05	0				0
539.25008	3.55E+04	0				0
539.32452	1.67E+04	0				0
540.33668	4.59E+04	0				0

540.90743	2.59E+05	$C_{15}H_{10}O_{16}S_3$	[M-H]⁻	540.90583	2.96	["Quercetin 3,3′,7-trissulfate", "Quercetin 3,4′,7-trissulfate"]
540.97531	7.60E+05	0				0
541.91091	2.08E+04	0				0
541.92578	3.62E+04	0				0
541.97865	8.60E+04	$C_{12}H_{20}N_4O_{10}P_3S$	$[M + (^{37}Cl)]^-$	541.97776	1.64	['Thiamin triphosphate']
543.22406	5.35E+04	$C_{29}H_{36}O_{10}$	[M-H]⁻	543.22357	0.9	['10-Deacetylbaccatin III']
543.23781	5.95E+04	$C_{29}H_{38}N_4O_4$	[M + K-2H]⁻	543.23791	−0.19	['Mucronine A']
543.23781	5.95E+04	$C_{33}H_{36}O_7$	[M-H]⁻	543.23883	−1.88	['Morellin']
543.25991	3.88E+04	0				0
543.94984	2.39E+05	0				0
545.23940	6.63E+04	0				0
545.27481	3.01E+04	$C_{30}H_{42}O_9$	[M-H]⁻	545.27561	−1.46	['Decoside']
546.82511	2.86E+04	0				0
546.92438	9.97E+04	0				0
547.21847	5.98E+04	$C_{26}H_{32}O_9$	[M + HAc-H]⁻	547.21849	−0.03	['Ichangin', 'Limonoate A-ring-lactone', 'Limonoate D-ring-lactone']
547.21847	5.98E+04	$C_{28}H_{36}O_{11}$	[M-H]⁻	547.21849	−0.03	['Bruceantin']
547.28833	9.78E+03	0				0
547.31255	1.08E+04	0				0
549.27091	3.91E+04	$C_{29}H_{42}O_{10}$	[M-H]⁻	549.27052	0.7	['Adonitoxin', 'Aspecioside', 'Convallatoxin']
550.84501	2.84E+04	0				0
551.28734	8.25E+04	$C_{28}H_{36}N_4O_4$	[M + HAc-H]⁻	551.28751	−0.31	['Mucronine B']
551.28734	8.25E+04	$C_{29}H_{44}O_{10}$	[M-H]⁻	551.28617	2.12	['Antioside', 'Bipindoside', 'Lokundjoside', 'Panogenin-3-*O*-alpha-L-rhamnopyranoside']
551.32280	8.12E+03	$C_{31}H_{44}N_4O_5$	[M-H]⁻	551.32389	−1.99	['Pandamine']
552.30035	4.46E+04	0				0
552.89525	2.42E+04	0				0
553.26578	2.77E+04	0				0
554.31611	6.33E+04	0				0
555.25971	1.35E+04	0				0

556.33161	4.44E+04	0				0
556.88258	8.72E+04	0				0
556.94910	2.09E+04	0				0
557.23984	4.18E+04	$C_{28}H_{34}O_8$	$[M + HAc-H]^-$	557.23922	1.11	['Drummondin A']
557.33680	7.88E+05	0				0
557.34583	1.33E+04	0				0
558.34020	2.11E+05	0				0
558.87960	2.80E+04	0				0
559.25468	4.67E+04	0				0
559.33343	3.55E+04	0				0
560.90209	3.97E+04	0				0
563.00435	1.95E+04	0				0
563.16194	3.41E+04	0				0
564.28965	1.36E+04	0				0
564.89658	1.10E+04	0				0
565.30240	1.72E+04	$C_{29}H_{38}N_4O_4$	$[M + HAc-H]^-$	565.30316	−1.34	['Mucronine A']
567.28203	8.29E+04	$C_{29}H_{44}O_{11}$	$[M-H]^-$	567.28109	1.66	['Sarmentoloside']
568.28572	2.18E+04	0				0
568.29528	1.09E+04	0				0
570.22759	2.68E+04	0				0
570.31115	5.47E+03	0				0
572.22550	9.02E+03	0				0
572.24338	3.68E+05	0				0
573.24674	9.63E+04	$C_{29}H_{44}O_9$	$[M + K-2H]^-$	573.24714	−0.7	['Coroglaucigenin-3-*O*-alpha-L-rhamnopyranoside', 'Mallogenin-3-*O*-alpha-L-rhamnopyranoside', 'Rhodexin A']
573.24674	9.63E+04	$C_{33}H_{36}N_4O_4$	$[M + Na-2H]^-$	573.24832	−2.76	['Canthiumine']
573.33175	9.85E+05	0				0
573.84420	2.57E+04	0				0
574.24036	1.23E+05	0				0
574.33513	2.67E+05	0				0

574.88948	5.33E+04	0				0
575.28662	4.32E+04	$C_{29}H_{40}O_8$	[M + HAc-H]⁻	575.28617	0.78	[‘Ajugalactone’]
576.28994	1.22E+04	0				0
576.93976	4.51E+04	0				0
577.00545	2.15E+04	$C_{18}H_{18}N_6O_8S_3$	[M + Cl]⁻	577.00423	2.11	[‘Cefonicid’]
577.30227	2.85E+04	$C_{30}H_{38}N_4O_4$	[M + HAc-H]⁻	577.30316	−1.54	[‘Sativanine B’]
577.30227	2.85E+04	$C_{31}H_{46}O_{10}$	[M-H]⁻	577.30182	0.77	[‘Oscillatoxin A’]
577.86413	2.24E+04	0				0
579.86123	1.52E+04	0				0
580.92708	7.99E+04	0				0
582.27227	4.80E+04	$C_{33}H_{37}N_5O_5$	[M-H]⁻	582.27219	0.13	[‘Dihydroergotamine’]
587.26403	3.35E+04	$C_{29}H_{44}O_{10}$	[M + Cl]⁻	587.26285	2.01	[‘Antioside’, ‘Bipindoside’, ‘Lokundjoside’, ‘Panogenin-3-o-alpha-L-rhamnopyranoside’]
590.79683	1.72E+04	0				0
591.28150	3.05E+04	$C_{29}H_{40}O_9$	[M + HAc-H]⁻	591.28109	0.7	[‘Calactin’, ‘Calotropin’, ‘Roridin A’]
592.35761	6.70E+03	0				0
593.29715	2.45E+04	$C_{29}H_{42}O_9$	[M + HAc-H]⁻	593.29674	0.69	[‘Corotoxigenin-3-*O*-alpha-L-rhamnopyranoside’, ‘Helveticoside’]
593.92071	6.57E+04	0				0
594.27220	4.27E+04	0				0
594.37311	5.83E+03	0				0
595.10457	3.05E+04	0				0
595.27614	2.34E+04	0				0
595.29261	2.88E+04	$C_{34}H_{44}O_9$	[M-H]⁻	595.29126	2.27	[‘Salannin’]
595.34947	8.50E+03	$C_{33}H_{48}N_4O_6$	[M-H]⁻	595.35011	−1.07	[‘L-Urobilinogen’]
596.28790	6.34E+05	0				0
596.81342	9.98E+03	0				0
597.29138	1.91E+05	0				0
598.29525	2.59E+04	$C_{21}H_{41}N_5O_{11}$	[M + HAc-H]⁻	598.29411	1.9	[‘Apramycin’]
598.29525	2.59E+04	$C_{23}H_{45}N_5O_{13}$	[M-H]⁻	598.29411	1.9	[‘Lividomycin B’]
598.81064	5.72E+03	0				0

598.86563	2.56E+04	0				0
598.93402	1.33E+05	0				0
599.26235	9.31E+04	$C_{30}H_{44}O_{10}$	[M + Cl]⁻	599.26285	−0.84	[‘Musaroside’, ‘Vernadigin’]
599.30677	2.01E+04	0				0
600.26577	2.72E+04	0				0
600.98400	1.04E+05	0				0
601.36316	4.54E+05	$C_{40}H_{52}O_2$	[M + (37Cl)]⁻	601.36318	−0.04	[‘Canthaxanthin’]
601.90859	5.23E+04	0				0
602.36652	1.35E+05	0				0
602.44649	7.40E+04	0				0
603.28131	1.81E+04	$C_{32}H_{44}O_{11}$	[M-H]⁻	603.28109	0.37	[‘Cerbertin’, ‘Lanceotoxin B’]
603.95819	3.96E+04	0				0
605.92038	4.21E+04	0				0
608.35244	7.96E+03	0				0
608.82428	3.49E+04	0				0
608.96289	2.38E+04	0				0
609.32856	1.39E+04	0				0
610.26640	1.33E+04	0				0
611.28716	2.78E+04	$C_{33}H_{36}N_4O_4$	[M + HAc-H]⁻	611.28751	−0.57	[‘Canthiumine’]
611.38058	2.51E+04	0				0
612.25293	1.24E+05	0				0
612.27118	2.47E+03	0				0
613.22988	1.38E+04	$C_{30}H_{34}O_{10}$	[M + HAc-H]⁻	613.22905	1.35	[‘Lappaol C’]
613.22988	1.38E+04	$C_{36}H_{36}N_2O_5$	[M + (^{37}Cl)]⁻	613.22887	1.64	[‘Tiliacorine’]
613.30306	5.08E+04	$C_{32}H_{42}O_8$	[M + HAc-H]⁻	613.30182	2.02	[‘Acrovestone’]
613.30306	5.08E+04	$C_{33}H_{38}N_4O_4$	[M + HAc-H]⁻	613.30316	−0.16	[‘Integerressine’]
613.92236	1.32E+04	0				0
614.84118	3.95E+04	0				0
616.83825	1.81E+04	0				0
617.35798	7.05E+05	$C_{40}H_{52}O_3$	[M + (^{37}Cl)]⁻	617.35810	−0.19	[‘Phoenicoxanthin’]
617.40615	7.87E+03	0				0

618.36140	2.13E+05	0				0
620.85784	1.67E+04	0				0
620.93942	4.47E+04	0				0
621.32847	1.05E+04	0				0
622.91016	5.47E+04	0				0
622.97844	1.28E+05	0				0
623.38065	7.96E+03	0				0
625.95298	4.89E+04	0				0
626.83122	2.08E+04	0				0
627.31763	1.33E+04	$C_{33}H_{44}O_8$	[M + HAc-H]⁻	627.31747	0.25	[“5-Oxoavermectin ‘’1b’’ aglycone”]
627.31763	1.33E+04	$C_{34}H_{40}N_4O_4$	[M + HAc-H]⁻	627.31881	−1.88	[‘Adouetine Y’, ‘Crenatine A’, ‘Protoporphyrinogen IX’]
627.33807	2.95E+04	0				0
628.82825	2.06E+04	0				0
630.20202	5.48E+04	0				0
631.29006	2.75E+04	$C_{32}H_{50}O_{10}$	[M + K-2H]⁻	631.28901	1.67	[‘13-Deoxytedanolide’]
631.29006	2.75E+04	$C_{33}H_{46}N_4O_6$	[M + K-2H]⁻	631.29034	−0.45	[‘L-Urobilin’]
633.06804	7.29E+04	0				0
636.88827	5.80E+04	0				0
638.88540	6.30E+04	0				0
641.38287	1.97E+04	0				0
643.16808	4.58E+04	0				0
644.90238	2.27E+04	0				0
645.38957	2.44E+05	0				0
646.39287	7.87E+04	0				0
647.02225	3.14E+05	0				0
647.30748	1.42E+04	$C_{33}H_{40}N_4O_6$	[M + HAc-H]⁻	647.30864	−1.79	[‘D-Urobilin’]
648.22980	4.74E+04	0				0
650.87590	7.84E+04	0				0
653.39143	1.35E+04	0				0
654.24664	1.11E+05	0				0
655.25014	3.42E+04	0				0

655.84732	2.03E+04	0				0
656.24342	4.01E+04	0				0
659.08019	3.34E+04	0				0
659.86739	1.25E+04	0				0
660.93272	9.75E+04	0				0
661.38432	4.20E+05	$C_{42}H_{56}O_4$	$[M + (^{37}Cl)]^-$	661.38431	0.01	['2,2-Diketospirilloxanthin']
661.91704	8.84E+03	0				0
664.27595	2.55E+04	0				0
666.10995	9.90E+04	0				0
667.37081	1.41E+04	0				0
669.07552	2.55E+04	0				0
671.87798	9.40E+04	0				0
674.79712	1.18E+04	0				0
676.33762	1.20E+05	0				0
676.91734	1.03E+05	0				0
676.97029	1.24E+05	0				0
677.34145	3.89E+04	0				0
677.42524	3.06E+04	0				0
677.89479	1.15E+05	0				0
678.29101	2.65E+05	0				0
678.81683	9.10E+03	0				0
679.29464	8.37E+04	0				0
683.15277	2.21E+04	0				0
683.91176	2.96E+04	0				0
687.92367	2.51E+04	0				0
689.41569	2.28E+05	0				0
690.41895	7.74E+04	0				0
693.13752	3.47E+05	0				0
694.14105	8.14E+04	0				0
695.30674	2.81E+04	0				0
696.84401	2.69E+04	0				0

698.89638	5.31E+04	0				0
699.36135	2.57E+04	$C_{35}H_{56}O_{14}$	[M-H]⁻	699.35973	2.31	['Chalcomycin']
701.12684	5.37E+04	0				0
704.91329	2.84E+04	0				0
704.98167	3.03E+04	0				0
705.41055	3.31E+05	0				0
709.13269	3.11E+05	0				0
710.13596	6.57E+04	0				0
712.29856	1.14E+04	$C_{35}H_{43}NO_{11}$	[M + HAc-H]⁻	712.29747	1.53	['Rifamycin W-hemiacetal']
721.16920	7.17E+04	0				0
726.93091	1.49E+06	0				0
727.93421	2.06E+05	0				0
728.92648	4.66E+05	0				0
730.23317	6.41E+04	0				0
735.11144	5.81E+04	0				0
736.24979	6.67E+04	0				0
741.10632	1.07E+04	0				0
743.17382	1.70E+05	0				0
749.43700	1.83E+05	$C_{46}H_{64}O_6$	[M + (^{37}Cl)]⁻	749.43674	0.34	['Hydroxychlorobactene glucoside']
750.44034	6.81E+04	0				0
753.15860	3.80E+05	0				0
754.27754	1.38E+05	0				0
755.94893	8.42E+06	0				0
756.95074	1.26E+06	0				0
757.95344	1.45E+05	0				0
760.29406	9.56E+04	0				0
761.29683	3.25E+04	$C_{36}H_{52}O_{15}$	[M + (^{37}Cl)]⁻	761.29708	−0.32	['Hellebrin']
762.92827	1.77E+05	0				0
765.44381	2.93E+04	$C_{41}H_{66}O_{13}$	[M-H]⁻	765.44307	0.97	['Akeboside Std', 'Tautomycin']
772.13705	3.49E+04	0				0
778.52482	2.82E+04	0				0

793.46327	9.85E+04	0				0
794.46650	3.89E+04	0				0

Table S3. Putative annotations of metabolites measured in the C18 stickleback class using FT-ICR mass spectrometry.

m/*z*	Median Intensity	Empirical Formula	Ion Form	Theoretical Mass (Da)	Mass Error (ppm)	KEGG_COMPOUND
122.97568	4.23E+03	$C_2H_4O_4S$	$[M\text{-}H]^-$	122.97576	−0.61	['Sulfoacetaldehyde']
122.97568	4.23E+03	O_2S	$[M + HAc\text{-}H]^-$	122.97576	−0.61	['Sulfur dioxide']
138.97059	3.00E+03	$C_2H_4O_5S$	$[M\text{-}H]^-$	138.97067	−0.58	['Sulfoacetate']
186.03122	1.56E+04	0				0
212.99137	1.04E+03	$C_5H_8N_2O_5$	$[M + K\text{-}2H]^-$	212.99193	−2.63	['*N*-Carbamoyl-L-aspartate', 'N_3-Oxalyl-L-2,3-diaminopropanoate']
216.08550	1.06E+03	$C_6H_{18}N_3OP$	$[M + (^{37}Cl)]^-$	216.08520	1.38	['Hexamethylphosphoramide']
265.15104	1.13E+05	0				0
293.17939	3.07E+06	0				0
293.18294	8.50E+04	0				0
294.18275	5.45E+05	$C_{13}H_{21}N_3O$	$[M + HAc\text{-}H]^-$	294.18232	1.48	['Procainamide']
295.17519	1.18E+05	0				0
296.17859	1.89E+04	$C_{17}H_{27}NO$	$[M + Cl]^-$	296.17867	−0.26	['Cryptophorine']
296.17859	1.89E+04	$C_{18}H_{29}N$	$[M + K\text{-}2H]^-$	296.17861	−0.05	['4Z,7Z,10Z-Octadecatrienenitrile']
307.19507	1.93E+04	0				0
309.17432	3.02E+06	$C_{17}H_{26}N_2O$	$[M + Cl]^-$	309.17391	1.31	['Ropivacaine', 'Sauroxine', 'alpha-Obscurine']
309.17432	3.02E+06	$C_{18}H_{20}N$	$[M + HAc\text{-}H]^-$	309.17343	2.89	['*cis*-*N*-Methyl-(*S*)-7,8,13,14-tetrahydroprotoberberine']
310.17768	4.59E+05	0				0
321.21070	9.49E+05	0				0
322.21405	1.58E+05	$C_{15}H_{25}N_3O$	$[M + HAc\text{-}H]^-$	322.21362	1.35	['Triapenthenol']
323.10676	1.12E+05	$C_{15}H_{20}N_2O_4S$	$[M\text{-}H]^-$	323.10710	−1.06	['Acetohexamide', 'Chromanol 293B', 'HMR1556']
323.20652	4.27E+04	0				0
337.20564	3.15E+06	0				0
337.21000	1.85E+05	0				0
338.20899	5.82E+05	0				0

343.01723	2.75E+04	0				0
350.12574	1.01E+05	0				0
353.05250	8.74E+04	0				0
353.20052	5.45E+06	0				0
354.20393	9.22E+05	0				0
354.20838	5.78E+04	$C_{20}H_{33}N_2O$	$[M + K-2H]^-$	354.20789	1.37	['Hexocyclium']
354.20838	5.78E+04	$C_{22}H_{29}NO_3$	$[M-H]^-$	354.20747	2.58	['Spirasine I']
355.19644	2.15E+05	$C_{14}H_{30}N_4O_5$	$[M + Na-2H]^-$	355.19629	0.42	['Fortimicin AP']
356.19981	3.57E+04	0				0
362.20097	1.49E+04	0				0
365.23700	7.23E+05	0				0
366.24037	1.45E+05	$C_{22}H_{35}NO_2$	$[M + Na-2H]^-$	366.24145	−2.94	['Himbacine']
367.13307	8.25E+04	0				0
378.15705	5.90E+04	0				0
381.23191	2.58E+06	0				0
382.23525	5.37E+05	0				0
383.23571	2.42E+04	$C_{22}H_{36}O_3$	$[M + Cl]^-$	383.23585	−0.36	['17beta-Hydroxy-2alpha-(methoxymethyl)-17-methyl-5alpha-androstan-3-one', 'Anacardic acid']
383.23571	2.42E+04	$C_{23}H_{38}O_2$	$[M + K-2H]^-$	383.23579	−0.2	['5-(Heptadec-12-enyl)resorcinol']
383.23571	2.42E+04	$C_{26}H_{34}O$	$[M + Na-2H]^-$	383.23563	0.2	['3-(2,4-Cyclopentadien-1-ylidene)pregn-4-en-20-one']
389.07687	3.47E+04	$C_{16}H_{20}N_2O_5S$	$[M + (^{37}Cl)]^-$	389.07575	2.89	['Benzylpenicilloic acid']
391.17739	1.33E+05	$C_{19}H_{24}O_5$	$[M + HAc-H]^-$	391.17623	2.97	['Gibberellin A20', 'Gibberellin A4', 'Gibberellin A51', 'Trichothecin']
391.17739	1.33E+05	$C_{21}H_{28}O_7$	$[M-H]^-$	391.17623	2.97	['Picrasin G', 'Viguiestenin']
393.15588	9.13E+04	$C_{20}H_{26}O_8$	$[M-H]^-$	393.15549	0.98	['Glaucarubolone', 'Specionin']
394.15205	6.35E+04	0				0
397.15038	5.15E+04	$C_{17}H_{22}O_7$	$[M + HAc-H]^-$	397.15041	−0.07	['1-Peroxyferolide']
397.22684	3.65E+06	0				0
398.23021	7.11E+05	$C_{22}H_{35}NO_4$	$[M + Na-2H]^-$	398.23128	−2.68	['Karakoline']
399.09302	3.02E+04	$C_{13}H_{22}N_4O_8$	$[M + K-2H]^-$	399.09237	1.62	['Clavamycin B', 'Clavamycin C']
399.09302	3.02E+04	$C_{15}H_{16}O_9$	$[M + HAc-H]^-$	399.09329	−0.67	['Cichoriin', 'Daphnin', 'Esculin', 'Sinapoyl malate']

399.22266	1.55E+05	0				0
409.26325	6.99E+05	0				0
410.26657	1.50E+05	0				0
417.89833	1.81E+04	0				0
425.25816	2.12E+06	0				0
426.26151	4.66E+05	$C_{24}H_{39}NO_4$	[M + Na-2H]⁻	426.26258	−2.5	['Cassaine']
427.12425	1.66E+04	$C_{17}H_{20}O_9$	[M + HAc-H]⁻	427.12459	−0.79	['*O*-Feruloylquinate']
428.25729	2.21E+04	0				0
435.20359	1.60E+05	$C_{21}H_{28}O_6$	[M + HAc-H]⁻	435.20244	2.63	['Picrasin B', 'Rhipocephalin']
435.20359	1.60E+05	$C_{23}H_{32}O_8$	[M-H]⁻	435.20244	2.63	['Polhovolide']
436.20689	2.99E+04	$C_{24}H_{33}NO_4$	[M + (^{37}Cl)]⁻	436.20741	−1.19	['Butroxydim', 'Spiramine A']
437.11511	2.64E+04	0				0
438.17818	6.58E+04	0				0
439.19061	6.78E+04	0				0
440.19367	2.02E+04	$C_{19}H_{27}NO_7$	[M + HAc-H]⁻	440.19261	2.41	['Petasitenine']
440.19367	2.02E+04	$C_{25}H_{29}N_3O_2$	[M + (^{37}Cl)]⁻	440.19243	2.82	['Pleurostyline']
442.26184	1.95E+04	0				0
443.11915	2.48E+04	0				0
443.24887	1.24E+05	0				0
451.16879	4.96E+04	0				0
451.32925	2.22E+05	$C_{27}H_{46}N_2O_2$	[M + Na-2H]⁻	451.33060	−2.98	['Solanocapsine']
453.28947	6.26E+05	0				0
454.29274	1.53E+05	$C_{26}H_{43}NO_4$	[M + Na-2H]⁻	454.29388	−2.5	['Glycolithocholate']
454.91647	4.10E+04	0				0
455.18543	1.35E+05	$C_{24}H_{34}O_4S$	[M + (^{37}Cl)]⁻	455.18423	2.63	['17beta-Hydroxy-4-mercaptoandrost-4-en-3-one 4-acetate 17-propionate']
455.18543	1.35E+05	$C_{24}H_{34}O_6$	[M + K-2H]⁻	455.18415	2.82	['11beta,17,21-Trihydroxy-2alpha-methylpregn-4-ene-3,20-dione 21-acetate', '21-Acetoxy-11beta,17-dihydroxy-6alpha-methylpregn-4-ene-3,20-dione', 'Phyllanthin']
455.18543	1.35E+05	$C_{25}H_{30}N_4O_2$	[M + K-2H]⁻	455.18548	−0.12	['Naphthyl dipeptide']
455.28522	3.31E+04	0				0

455.29165	4.71E+03	0				0
463.23493	1.64E+05	$C_{23}H_{32}O_6$	[M + HAc-H]⁻	463.23374	2.56	['Cortisol 21-acetate']
463.23493	1.64E+05	$C_{25}H_{36}O_8$	[M-H]⁻	463.23374	2.56	['Testosterone glucuronide']
466.20941	7.09E+04	0				0
467.16361	6.81E+04	$C_{27}H_{28}O_5$	[M + Cl]⁻	467.16308	1.14	['Aspulvinone H']
467.22185	3.97E+04	$C_{25}H_{36}O_6$	[M + Cl]⁻	467.22059	2.69	['Glycinoeclepin A']
468.16690	1.90E+04	0				0
469.28434	2.03E+06	0				0
470.13811	1.87E+04	0				0
470.28171	2.52E+04	0				0
470.28768	4.83E+05	$C_{26}H_{43}NO_5$	[M + Na-2H]⁻	470.28879	−2.37	['3alpha,12alpha-Dihydroxy-5beta-cholan-24-oylglycine', 'Glycochenodeoxycholate', 'Glycodeoxycholate'] .
471.28015	9.75E+04	0				0
471.28680	1.82E+04	0				0
472.28354	2.47E+04	0				0
479.22985	2.37E+05	0				0
480.23313	5.69E+04	0				0
483.21667	9.22E+04	$C_{18}H_{36}N_6O_5S$	[M + Cl]⁻	483.21619	0.99	['Glutathionylaminopropylcadaverine']
485.27930	2.45E+06	0				0
486.28263	5.70E+05	$C_{26}H_{43}NO_6$	[M + Na-2H]⁻	486.28371	−2.22	['Glycocholate']
487.27524	1.12E+05	0				0
487.36756	5.44E+04	0				0
488.27850	2.82E+04	$C_{25}H_{43}NO_6$	[M + Cl]⁻	488.27844	0.12	['YC-17']
488.27850	2.82E+04	$C_{29}H_{41}NO_4$	[M + Na-2H]⁻	488.27823	0.56	['Buprenorphine']
493.16241	4.09E+04	$C_{26}H_{32}O_7$	[M + K-2H]⁻	493.16341	−2.03	['Kuraridinol', 'Kurarinol']
495.19482	4.09E+04	0				0
496.19805	1.55E+04	0				0
497.31568	4.54E+05	0				0
499.21174	1.05E+05	$C_{25}H_{36}O_8$	[M + Cl]⁻	499.21042	2.64	['Testosterone glucuronide']
499.21174	1.05E+05	$C_{26}H_{38}O_7$	[M + K-2H]⁻	499.21036	2.76	['10-Desacetyltaxuyunnanin C']
500.21504	2.45E+04	0				0

502.31753	3.73E+04	0				0
507.26119	1.02E+05	$C_{18}H_{36}N_6O_5S$	[M + HAc-H]⁻	507.26064	1.08	[‘Glutathionylaminopropylcadaverine’]
510.23577	4.11E+04	0				0
511.18984	6.17E+04	0				0
513.31070	1.24E+06	0				0
514.31407	3.32E+05	0				0
515.17674	2.19E+04	$C_{20}H_{22}N_7O_6$	[M + HAc-H]⁻	515.17701	−0.53	[‘5,10-Methenyltetrahydrofolate’]
525.27063	2.11E+04	$C_{25}H_{38}O_8$	[M + HAc-H]⁻	525.27052	0.2	[‘Androsterone glucuronide’, ‘Etiocholan-3alpha-ol-17-one 3-glucuronide’]
526.23065	5.72E+04	0				0
527.24308	6.70E+04	$C_{28}H_{36}N_4O_4$	[M + Cl]⁻	527.24306	0.04	[‘Mucronine B’]
528.24637	1.93E+04	0				0
529.30554	1.96E+06	0				0
530.30887	5.05E+05	$C_{28}H_{47}NO_7$	[M + Na-2H]⁻	530.30992	−1.98	[‘Narbomycin’]
530.31683	9.76E+03	0				0
535.29220	5.83E+04	$C_{27}H_{40}O_7$	[M + HAc-H]⁻	535.29126	1.76	[‘Cyclic-3,20-bis(1,2-ethanediyl acetal)-11alpha-(acetyloxy)-5alpha,6alpha-epoxypregnane-3,20-dione’]
535.29220	5.83E+04	$C_{29}H_{44}O_9$	[M-H]⁻	535.29126	1.76	[‘Coroglaucigenin-3-o-alpha-L-rhamnopyranoside’, ‘Mallogenin-3-o-alpha-L-rhamnopyranoside’, ‘Rhodexin A’]
541.34189	3.33E+05	0				0
542.34531	8.92E+04	0				0
543.23784	7.77E+04	$C_{29}H_{38}N_4O_4$	[M + K-2H]⁻	543.23791	−0.13	[‘Mucronine A’]
543.23784	7.77E+04	$C_{33}H_{36}O_7$	[M-H]⁻	543.23883	−1.82	[‘Morellin’]
544.24122	2.19E+04	0				0
551.28737	1.10E+05	$C_{28}H_{36}N_4O_4$	[M + HAc-H]⁻	551.28751	−0.25	[‘Mucronine B’]
551.28737	1.10E+05	$C_{29}H_{44}O_{10}$	[M-H]⁻	551.28617	2.17	[‘Antioside’, ‘Bipindoside’, ‘Lokundjoside’, ‘Panogenin-3-*O*-alpha-L-rhamnopyranoside’]
554.26179	3.92E+04	0				0
555.21610	8.28E+04	0				0
556.21926	2.99E+04	0				0

557.33679	1.00E+06	0				0
557.34576	1.96E+04	0				0
558.34018	2.70E+05	0				0
559.34349	3.62E+04	0				0
567.28230	1.08E+05	$C_{29}H_{44}O_{11}$	[M-H]⁻	567.28109	2.14	['Sarmentoloside']
568.28559	2.73E+04	0				0
570.25684	3.34E+04	0				0
571.26917	4.27E+04	$C_{29}H_{44}O_{9}$	$[M + Cl]^-$	571.26794	2.16	['Coroglaucigenin-3-*O*-alpha-L-rhamnopyranoside', 'Mallogenin-3-*O*-alpha-L-rhamnopyranoside', 'Rhodexin A']
571.26917	4.27E+04	$C_{30}H_{46}O_{8}$	$[M + K-2H]^-$	571.26788	2.26	['Cucurbitacin H', 'Divaricoside', 'Divostroside', 'Neriifolin']
571.26917	4.27E+04	$C_{31}H_{42}N_{4}O_{4}$	$[M + K-2H]^-$	571.26921	−0.07	['Integerrenine']
573.33173	1.28E+06	0				0
573.34114	2.34E+04	0				0
574.33513	3.43E+05	0				0
584.40982	2.91E+04	0				0
585.36826	1.84E+05	0				0
585.98980	2.57E+04	0				0
587.14388	4.33E+04	0				0
593.31330	1.68E+04	0				0
595.00789	4.40E+04	0				0
595.31361	5.96E+04	$C_{29}H_{44}O_{9}$	$[M + HAc-H]^-$	595.31239	2.05	['Coroglaucigenin-3-*O*-alpha-L-rhamnopyranoside', 'Mallogenin-3-*O*-alpha-L-rhamnopyranoside', 'Rhodexin A']
601.36310	6.77E+05	$C_{40}H_{52}O_{2}$	$[M + (^{37}Cl)]^-$	601.36318	−0.14	['Canthaxanthin']
601.37338	1.10E+04	0				0
602.36653	1.99E+05	0				0
607.34959	3.17E+04	0				0
613.17485	2.04E+04	0				0
615.29512	2.79E+04	0				0

617.35797	9.59E+05	$C_{40}H_{52}O_3$	[M + (^{37}Cl)]$^-$	617.35810	−0.21	['Phoenicoxanthin']
618.36143	2.84E+05	0				0
627.33773	3.52E+04	0				0
629.39445	1.32E+05	0				0
630.39780	4.23E+04	0				0
637.21942	3.20E+04	0				0
645.38944	3.64E+05	0				0
646.39282	1.19E+05	0				0
657.34844	2.68E+04	0				0
660.97245	4.54E+04	0				0
661.38427	5.70E+05	$C_{42}H_{56}O_4$	[M + (^{37}Cl)]$^-$	661.38431	−0.06	['2,2-Diketospirilloxanthin']
662.38772	1.87E+05	0				0
665.10650	6.79E+05	0				0
666.10994	1.37E+05	0				0
671.36410	4.32E+04	0				0
673.35819	7.70E+04	$C_{37}H_{54}O_{11}$	[M-H]$^-$	673.35934	−1.71	['Cimicifugoside']
673.42074	8.51E+04	0				0
689.41564	3.12E+05	0				0
690.41896	1.05E+05	0				0
693.13766	4.99E+05	0				0
694.14116	1.02E+05	0				0
699.36101	3.53E+04	$C_{35}H_{56}O_{14}$	[M-H]$^-$	699.35973	1.83	['Chalcomycin']
701.46988	4.15E+04	0				0
705.41048	4.49E+05	0				0
706.41394	1.52E+05	0				0
709.13264	4.31E+05	0				0
710.13599	8.65E+04	0				0
717.44721	5.00E+04	0				0
734.44537	7.12E+04	0				0
740.16221	2.05E+05	0				0
749.43693	2.61E+05	$C_{46}H_{64}O_6$	[M + (^{37}Cl)]$^-$	749.43674	0.25	['Hydroxychlorobactene glucoside']

750.44042	9.62E+04	0				0
765.47807	2.80E+04	0				0
778.47156	4.48E+04	0				0
781.19022	1.50E+05	0				0
782.19376	4.28E+04	0				0
793.46308	1.44E+05	0				0
794.46650	5.77E+04	0				0

Table S4. Putative annotations of metabolites measured in SDB-RPS stickleback class using FT-ICR mass spectrometry.

m/z	Median Intensity	Empirical Formula	Ion Form	Theoretical Mass (Da)	Mass Error (ppm)	KEGG_COMPOUND
185.02788	1.33E+05	0				0
186.03125	1.33E+04	0				0
187.02370	7.48E+03	$C_6H_6N_4O_2$	$[M + Na\text{-}2H]^-$	187.02374	−0.24	['1-Methylxanthine', '3-Methylxanthine', '7-Methylxanthine']
200.04692	5.10E+03	0				0
201.03939	2.30E+03	$C_7H_8N_4O_2$	$[M + Na\text{-}2H]^-$	201.03939	−0.02	['1,7-Dimethylxanthine', 'Theobromine', 'Theophylline']
293.17942	2.54E+06	0				0
294.18279	4.43E+05	$C_{13}H_{21}N_3O$	$[M + HAc\text{-}H]^-$	294.18232	1.61	['Procainamide']
295.17522	1.01E+05	0				0
309.17436	2.59E+06	$C_{17}H_{26}N_2O$	$[M + Cl]^-$	309.17391	1.44	['Ropivacaine', 'Sauroxine', 'alpha-Obscurine']
310.17772	3.92E+05	0				0
321.21074	7.84E+05	0				0
322.21409	1.33E+05	$C_{15}H_{25}N_3O$	$[M + HAc\text{-}H]^-$	322.21362	1.47	['Triapenthenol']
325.10397	3.35E+04	$C_{12}H_{14}N_2O_5$	$[M + HAc\text{-}H]^-$	325.10413	−0.48	['Dinex']
325.10397	3.35E+04	$C_{14}H_{18}N_2O_7$	$[M\text{-}H]^-$	325.10413	−0.48	['Dinobuton', 'Humilixanthin']
337.20569	2.67E+06	0				0
337.20999	1.73E+05	0				0
338.20903	4.96E+05	0				0
353.20057	4.36E+06	0				0
354.20397	7.55E+05	0				0

355.19651	1.70E+05	$C_{14}H_{30}N_4O_5$	[M + Na-2H]⁻	355.19629	0.62	['Fortimicin AP']
356.19986	2.95E+04	0				0
365.23703	5.51E+05	0				0
366.24038	1.12E+05	$C_{22}H_{35}NO_2$	[M + Na-2H]⁻	366.24145	−2.92	['Himbacine']
367.13311	7.50E+04	0				0
369.98029	2.07E+04	0				0
381.19231	3.00E+04	$C_{18}H_{26}O_5$	[M + HAc-H]⁻	381.19188	1.13	['alpha-Zearalanol', 'beta-Zearalanol']
381.19231	3.00E+04	$C_{20}H_{30}O_7$	[M-H]⁻	381.19188	1.13	['Cinncassiol A', 'Cinncassiol C3']
381.23194	2.08E+06	0				0
383.22770	9.75E+04	0				0
398.23024	5.51E+05	$C_{22}H_{35}NO_4$	[M + Na-2H]⁻	398.23128	−2.61	['Karakoline']
399.18178	3.47E+04	$C_{21}H_{24}O_4$	[M + HAc-H]⁻	399.18131	1.17	['16beta-Hydroxy-3,11-dioxopregna-4,17(20)-dien-21-oic acid, gamma-lactone', 'Bisphenol A diglycidyl ether'] .
399.18178	3.47E+04	$C_{23}H_{28}O_6$	[M-H]⁻	399.18131	1.17	['Cortancyl', 'Deoxygomisin A', 'Gomisin L1 methyl ether']
399.22266	1.29E+05	0				0
399.23413	3.76E+04	0				0
409.26329	5.47E+05	0				0
412.96226	1.41E+04	0				0
415.17685	2.92E+04	$C_{21}H_{24}O_5$	[M + HAc-H]⁻	415.17623	1.5	['Denudatin B', 'Gingerenone A', 'Kadsurenone', 'Rutamarin']
415.17685	2.92E+04	$C_{23}H_{28}O_7$	[M-H]⁻	415.17623	1.5	['Erioflorin methacrylate']
419.95635	6.47E+04	0				0
425.25821	1.64E+06	0				0
426.26155	3.64E+05	$C_{24}H_{39}NO_4$	[M + Na-2H]⁻	426.26258	−2.41	['Cassaine']
427.25405	7.51E+04	0				0
453.28952	5.02E+05	0				0
455.18551	1.14E+05	$C_{24}H_{34}O_4S$	[M + (^{37}Cl)]⁻	455.18423	2.8	['17beta-Hydroxy-4-mercaptoandrost-4-en-3-one 4-acetate 17-propionate'] .
455.18551	1.14E+05	$C_{24}H_{34}O_6$	[M + K-2H]⁻	455.18415	3	['11beta,17,21-Trihydroxy-2alpha-methylpregn-4-ene-3,20-dione 21-acetate', '21-Acetoxy-11beta,17-dihydroxy-6alpha-methylpregn-4-ene-3,20-dione', 'Phyllanthin']

455.18551	1.14E+05	$C_{25}H_{30}N_4O_2$	[M + K-2H]⁻	455.18548	0.06	['Naphthyl dipeptide']
458.97828	2.21E+05	0				0
469.28438	1.79E+06	0				0
470.28172	2.37E+04	0				0
470.28770	4.29E+05	$C_{26}H_{43}NO_5$	[M + Na-2H]⁻	470.28879	−2.32	['3alpha,12alpha-Dihydroxy-5beta-cholan-24-oylglycine', 'Glycochenodeoxycholate', 'Glycodeoxycholate'] .
476.20084	2.46E+04	0				0
483.21654	7.85E+04	$C_{18}H_{36}N_6O_5S$	[M + Cl]⁻	483.21619	0.72	['Glutathionylaminopropylcadaverine']
485.27934	2.05E+06	0				0
487.27526	9.17E+04	0				0
496.27440	2.40E+05	$C_{21}H_{41}N_5O_7$	[M + Na-2H]⁻	496.27527	−1.75	['Netilmicin']
497.31579	3.78E+05	0				0
503.22910	5.33E+04	0				0
510.93424	8.30E+04	0				0
512.26927	3.52E+05	$C_{21}H_{41}N_5O_8$	[M + Na-2H]⁻	512.27018	−1.78	['N2-Acetylgentamicin C1a']
513.27267	9.90E+04	0				0
513.31069	9.42E+05	0				0
514.28479	4.78E+06	$C_{26}H_{45}NO_7S$	[M-H]⁻	514.28440	0.76	['Taurocholate']
515.28816	1.36E+06	0				0
516.28061	2.34E+05	$C_{25}H_{43}NO_{10}$	[M-H]⁻	516.28142	−1.57	['Mycalamide B']
530.30889	4.09E+05	$C_{28}H_{47}NO_7$	[M + Na-2H]⁻	530.30992	−1.95	['Narbomycin']
541.34198	2.87E+05	0				0
543.23778	6.80E+04	$C_{29}H_{38}N_4O_4$	[M + K-2H]⁻	543.23791	−0.24	['Mucronine A']
543.23778	6.80E+04	$C_{33}H_{36}O_7$	[M-H]⁻	543.23883	−1.93	['Morellin']
551.32286	9.19E+03	$C_{31}H_{44}N_4O_5$	[M-H]⁻	551.32389	−1.88	['Pandamine']
558.08510	1.40E+04	0				0
571.09754	5.06E+04	0				0
572.24341	4.24E+05	0				0
573.24643	1.09E+05	$C_{29}H_{44}O_9$	[M + K-2H]⁻	573.24714	−1.24	['Coroglaucigenin-3-o-alpha-L-rhamnopyranoside', 'Mallogenin-3-o-alpha-L-rhamnopyranoside', 'Rhodexin A']
590.88471	8.81E+03	0				0

596.28788	7.11E+05	0				0
597.29168	2.14E+05	0				0
601.36311	5.14E+05	$C_{40}H_{52}O_2$	[M + (^{37}Cl)]$^-$	601.36318	−0.12	[‘Canthaxanthin’]
607.34950	2.46E+04	0				0
618.36143	2.39E+05	0				0
630.20197	6.29E+04	0				0
632.19877	3.77E+04	0				0
639.08487	2.03E+04	0				0
641.17592	3.23E+04	0				0
654.24666	1.27E+05	0				0
655.33535	3.48E+04	0				0
656.95421	6.78E+04	0				0
661.38433	4.70E+05	$C_{42}H_{56}O_4$	[M + (^{37}Cl)]$^-$	661.38431	0.03	[‘2,2-Diketospirilloxanthin’]
665.10650	5.41E+05	0				0
666.10986	1.14E+05	0				0
678.29117	3.00E+05	0				0
683.22286	9.55E+04	0				0
689.41570	2.57E+05	0				0
694.14124	9.01E+04	0				0
705.41054	3.75E+05	0				0
710.13604	7.44E+04	0				0
713.47422	5.25E+04	0				0
726.93088	1.68E+06	0				0
727.93417	2.32E+05	0				0
760.29384	1.09E+05	0				0
776.78448	6.76E+03	0				0
776.80624	9.64E+03	0				0
776.81390	1.10E+04	0				0
776.84273	2.45E+04	0				0
776.85772	5.91E+04	0				0
776.90218	1.03E+06	0				0

776.95185	1.59E+06	0	0
777.00380	4.01E+04	0	0
777.93068	4.14E+07	0	0
778.93259	6.42E+06	0	0
779.93536	7.34E+05	0	0
781.19015	1.37E+05	0	0
793.46323	1.13E+05	0	0

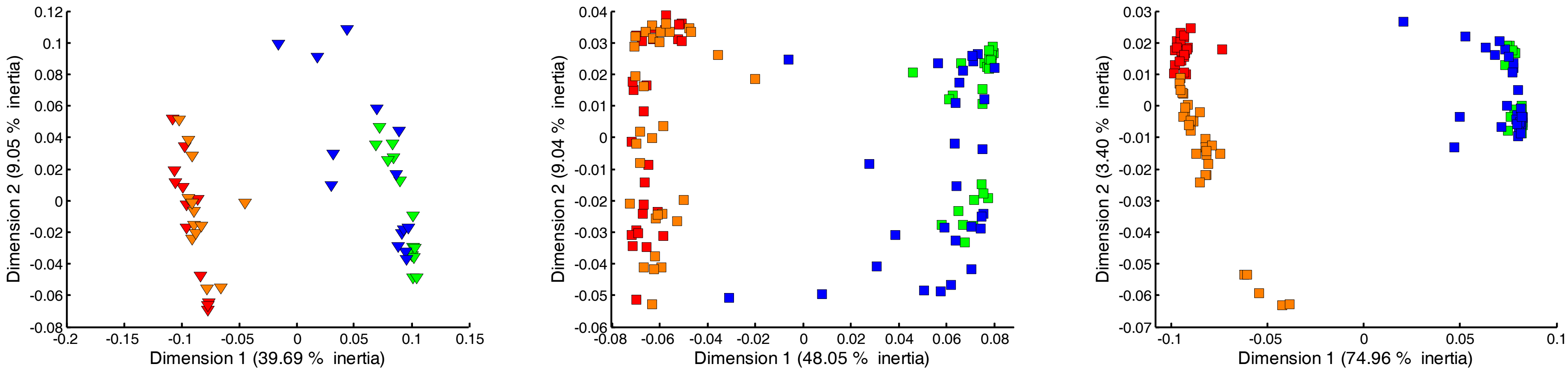

(**a**) (**b**) (**c**)

Figure S1. Multiple correspondence analyses of the metabolites captured on the Empore™ receiving phases in the fish aquaria and measured via mass spectrometry metabolomics (MANOVA, $p < 0.001$ for all three analyses): (**a**) C18 disk, trout (all pairwise comparisons between weeks 1–4 are significant ($p \leq 1.0\text{e-}18$), except week 1 vs. week 2 and week 3 vs. week 4); (**b**) SDB-RPS disk, stickleback (week 1 vs. week 2 is not significant ($p = 1$), week 3 vs. week 4 is significant ($p = 0.0014$), all other pairwise comparisons are highly significant ($p \leq 1\text{e-}33$)); (**c**) C18 disk, stickleback (all pairwise comparisons are significant ($p \leq 1.3\text{e-}10$), except week 3 vs. week 4). Key: metabolites captured in week 1 (red), week 2 (orange), week 3 (green) and week 4 (blue).
